# Root zone–specific localization of AMTs determines ammonium transport pathways and nitrogen allocation to shoots

**DOI:** 10.1371/journal.pbio.2006024

**Published:** 2018-10-24

**Authors:** Fengying Duan, Ricardo F. H. Giehl, Niko Geldner, David E. Salt, Nicolaus von Wirén

**Affiliations:** 1 Molecular Plant Nutrition, Leibniz Institute of Plant Genetics and Crop Plant Research, Corrensstr, Gatersleben, Germany; 2 Department of Plant Molecular Biology, Biophore, UNIL-Sorge, University of Lausanne, Lausanne, Switzerland; 3 Centre for Plant Integrative Biology, School of Biosciences, University of Nottingham, Sutton Bonington Campus, Loughborough, United Kingdom; University of Cambridge Sainsbury Laboratory, United Kingdom of Great Britain and Northern Ireland

## Abstract

In plants, nutrient provision of shoots depends on the uptake and transport of nutrients across the root tissue to the vascular system. Nutrient delivery to the vasculature is mediated via the apoplastic transport pathway (ATP), which uses the free space in the cell walls and is controlled by apoplastic barriers and nutrient transporters at the endodermis, or via the symplastic transport pathway (STP). However, the relative importance of these transport routes remains elusive. Here, we show that the STP, mediated by the epidermal ammonium transporter 1;3 (AMT1;3), dominates the radial movement of ammonium across the root tissue when external ammonium is low, whereas apoplastic transport controlled by AMT1;2 at the endodermis prevails at high external ammonium. Then, AMT1;2 favors nitrogen (N) allocation to the shoot, revealing a major importance of the ATP for nutrient partitioning to shoots. When an endodermal bypass was introduced by abolishing Casparian strip (CS) formation, apoplastic ammonium transport decreased. By contrast, symplastic transport was increased, indicating synergism between the STP and the endodermal bypass. We further establish that the formation of apoplastic barriers alters the cell type–specific localization of AMTs and determines STP and ATP contributions. These results show how radial transport pathways vary along the longitudinal gradient of the root axis and contribute to nutrient partitioning between roots and shoots.

## Author summary

Radial transport of nutrients from the soil to the vascular system of plant roots occurs via the symplastic transport pathway (STP) and apoplastic transport pathway (ATP). Nutrients move along the STP when crossing the plasma membrane of outer cells and moving to xylem through the cytoplasmic continuum formed by plasmodesmata. Nutrients following the ATP, in turn, initially move passively through the extracellular space but are eventually taken up by endodermal cells, in which Casparian strips (CSs) prevent further apoplastic movement. We assessed the contribution of these transport pathways to radial transport in roots and nutrient provision to shoots by expressing cell type–specific ammonium transporters in a CS-defective mutant. Our study reveals that i) symplastic transport is more efficient at low external ammonium supply; ii) when endodermal cells become sealed by the deposition of suberin lamellae, the expression of ammonium transporters shifts to cortical cells; and iii) apoplastic transport depends on a functional apoplastic barrier at the endodermis, favoring nitrogen (N) partitioning to shoots at high external ammonium.

## Introduction

A major function of plant roots is the uptake and subsequent translocation of nutrients from soil to above-ground plant organs. To reach the shoot, nutrients need first to be transported radially across the root tissue before entering the xylem for root-to-shoot translocation. Once nutrients cross the plasma membrane of root epidermal cells, they enter the symplastic pathway, on which they move through the cytoplasmic continuum via plasmodesmata from cell to cell until they arrive in the xylem [1]. Nutrients may also enter the free space and cell walls of epidermal and cortical cells and move passively along the apoplastic route, which ultimately becomes blocked by the Casparian strip (CS) at the endodermis [2], where lignin depositions in anticlinal walls form a physical barrier to prevent an endodermal bypass [3]. This barrier prevents further inward movement in the apoplast. To progress further, nutrients must enter endodermal cells via membrane proteins, thereby completing the apoplastic transport pathway (ATP). As both pathways require a membrane transporter–mediated step, we refer here to the ATP and the symplastic transport pathway (STP). In basal root zones, endodermal cells become suberized, i.e., coated at the inner cell walls with aliphatic polymers, which form another apoplastic barrier, preventing access of nutrients to the plasma membrane [4,5]. Endodermal bypass, i.e., unhindered radial movement through cell walls of the endodermis, is only possible where these apoplastic barriers are not yet formed, such as in the apical root zone. Although the concept of radial nutrient transport, as determined by the ATP and STP, is common to all plant physiology textbooks [6,7], it still remains largely based on coincidences and theoretical considerations, as the significance and quantitative share between these two transport pathways has not yet been characterized for any mineral element.

A key methodological aspect required to dissect successfully the contribution of different radial transport pathways is the ability to manipulate the integrity of root apoplastic barriers. Earlier attempts to generate small artificial bypasses by puncturing endodermal cells with microcapillary tubes provoked instable root pressure and wounding responses [8]. However, with the recent isolation of mutants with impaired CS formation, it is possible for the first time to investigate these transport pathways at the molecular level. Among all CS-defective mutants characterized so far, the most suitable mutant is *schengen3* (*sgn3*), since it exhibits strong and persistent CS defects but no enhanced or precocious accumulation of suberin [9]. SGN3, also known as GASSHO1 (GSO1), is a leucine-rich repeat receptor–like kinase that acts as a receptor for 2 tyrosine-sulfated peptides known as CS integrity factors 1 and 2 (CIF1 and CIF2) [10,11]. As SGN3 function cannot be replaced by another protein, a large endodermal bypass is created in *sgn3* roots, turning this mutant into a valuable tool to assess the contribution and physiological relevance of different radial transport pathways.

Ammonium (NH_4_^+^) is a major source of soil nitrogen (N) for plants and is transported radially through the root tissue. A dedicated set of NH_4_^+^ transporters belonging to the ammonium transporter/methylamine permease/Rhesus-type (AMT/MEP/Rh-type) protein family is responsible for membrane transport of ammoniacal N in plants. Arabidopsis roots express 5 AMT-type transporters. Short-term influx studies had shown that under N deficiency, which up-regulates transcript levels of all 5 *AMT* genes in roots, 90%–95% of the high-affinity uptake capacity of NH_4_^+^ is mediated by AMT1;1, AMT1;2, and AMT1;3 [12,13]. In the low-affinity (millimolar) range, the contribution of AMT1-type transporters to overall NH_4_^+^ uptake shrinks because other low-affinity transporters come into play. For instance, AMT2;1, the only member belonging to MEP/AMT2 subfamily, contributes 10%–25% to the overall ammonium uptake rate at high external ammonium concentrations [13]. Other low-affinity transporters, such as ammonium facilitator–type transporters or potassium (K^+^) channels [14,15], may further contribute to low-affinity ammonium transport, but their physiological function in planta still remains unclear. Interestingly, AMT1;1 and AMT1;3 reside primarily in the epidermis and are involved in NH_4_^+^ uptake for the early passage into the STP. In contrast, AMT1;2 is located primarily in the endodermis, suggesting that this transporter completes the ATP for NH_4_^+^ [12,16]. Thus, the distinct cell type–specific expression of individual NH_4_^+^ transporters in roots and the near absence of AMT-independent NH_4_^+^ uptake in the high-affinity range make NH_4_^+^ transport via AMTs ideally suited to study radial transport pathways in plant roots.

Here, we quantify the relative contribution of the ATP and STP to radial transport of ^15^N-labeled ammonium (^15^NH_4_^+^). We show that a leaky CS causing an extended endodermal bypass acts synergistically with the AMT1;3-mediated STP while competing with the ATP mediated by AMT1;2. Together with cell type–specific localization of AMT proteins along the longitudinal axis of the Arabidopsis root, our study provides a novel basis for an improved mechanistic understanding on the contribution of radial transport pathways to nutrient partitioning between roots and shoots.

## Results

### Impact of an extended endodermal bypass in *sgn3* for radial element transport in roots

**Fig 1:**
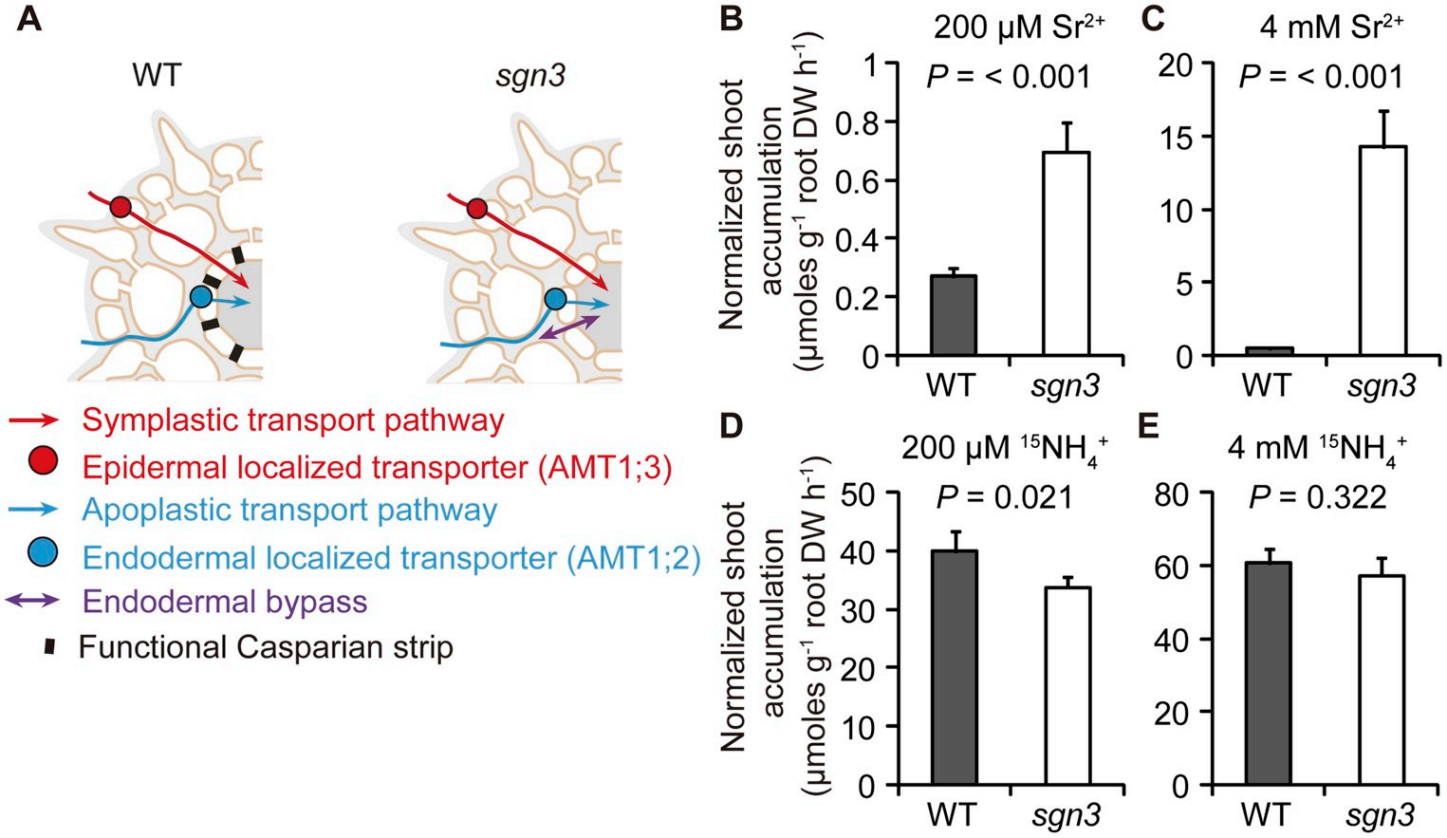
Role of the endodermal bypass in radial substrate transport in roots. (A) Schematic representation of radial transport pathways in roots with (WT, Columbia-0) or without (*sgn3* mutant) functional CSs. (B–E) Normalized shoot accumulation as readout for radial transport rates of Sr^2+^ or ^15^NH_4_^+^ when supplied in the nutrient solution at low (200 μM) or high (4 mM) concentration. Bars represent means ± SD. *P* values were calculated using Student *t* test (*n* = 4 biological replicates). Underlying data can be found in S1 Data. ^15^NH_4_^+^, ^15^N-labeled ammonium; CS, Casparian strip; DW, dry weight; *sgn3*, *schengen 3*; Sr^2+^, strontium ion; WT, wild-type.

The *sgn3* mutant was used to dissect the apoplastic route into an ATP, which entails an obligatory transporter-mediated step at the plasma membrane of the endodermis (Fig 1A, blue), and an endodermal bypass (Fig 1A, purple), allowing for unhindered diffusion across the endodermal layer. To quantify radial transport, we first exposed N-deficient plants to ^15^NH_4_^+^ and compared ^15^N accumulation in shoots to that in xylem exudates and found closely related patterns of ^15^N accumulation (S1 Fig). Since NH_4_^+^ uptake rates and the expression of *AMT*s increase during daytime and decrease in the dark [17], we assessed shoot accumulation rates during the light period, when transpiration is high and root pressure low, thereby suppressing the effect of root pressure on xylem transport rates. In order to favor transpiration as the major driving force for radial transport of water and nutrients, we calculated radial transport rates based on tracer accumulation in shoots rather than in xylem saps. Referring tracer accumulation in shoots to root dry weight and time (“normalized shoot accumulation”) thus integrates the rates of radial transport and of xylem loading in roots. Since AMT2;1, a critical transporter for root-to-shoot NH_4_^+^ translocation [13], was present in all tested lines, we assumed no significant changes in xylem loading and hence considered “normalized shoot accumulation” as a readout for radial substrate transport rates in roots.

We then verified the relevance of an extended endodermal bypass in *sgn3* by exposing hydroponically grown wild-type (WT) and *sgn3* mutant plants to strontium ion (Sr^2+^), which, similar to calcium ion (Ca^2+^), is transported to shoots mainly via the ATP [18–21]. Indeed, compared to wild type, normalized shoot accumulation of Sr^2+^ was approximately 3- or 20-fold higher in *sgn3* at low or high external Sr^2+^, respectively (Fig 1B and 1C). In roots, Sr^2+^ accumulation increased comparatively little (S2A and S2B Fig), indicating an extraordinary impact of the endodermal bypass generated in the *sgn3* mutant to radial transport and delivery of Sr^2+^ to the shoot. In the case of NH_4_^+^, the impact of the endodermal bypass on shoot or root accumulation of NH_4_^+^ was insignificant (Fig 1D and 1E, S2C and S2D Fig), suggesting that the action of the whole set of dedicated NH_4_^+^ transporters in WT and *sgn3* plants dominated over the contribution of an endodermal bypass. Thus, the relevance of a purely apoplastic pathway in the form of an extended endodermal bypass for radial element transport depends on the substrate and the presence of transporter-mediated radial pathways.

In agreement with an earlier study showing that phenotypical changes in *sgn3* plants are highly dependent on the prevailing environmental conditions [9], this mutant had significantly less root and shoot biomass than the wild type (S3A and S3C Fig). Potential pleiotropic effects arising from the *sgn3* mutation were assessed by verifying the relation between biomass and normalized shoot accumulation of NH_4_^+^ in *esb1* and *myb36*, 2 other CS-defective mutants [4,5]. Both mutants showed a similar decrease in shoot and root dry weight to *sgn3* (S3A and S3C Fig), but their normalized shoot accumulation of ^15^N was 20%–25% lower and went along with increased root accumulation of ^15^N (S3B and S3D Fig). Thus, we concluded that radial transport rates of NH_4_^+^ were not primarily affected by plant biomass but rather by the properties of existing apoplastic barriers. Unlike *sgn3*, the CS defects of *esb1* and *myb36* are partially compensated for by a stronger and earlier suberization of endodermal cells [4,5], which makes endodermal transporters inaccessible for their substrates [21]. Thus, the decreased biomass of *sgn3* is not specific to the *sgn3* mutation but most likely the consequence of a leaky CS. To compensate for the differences in root dry weight, we normalized all shoot accumulation rates to root dry weight, as practiced in other transport studies [22, 23].

### Quantitative contribution of different pathways to radial ammonium transport in roots

**Fig 2:**
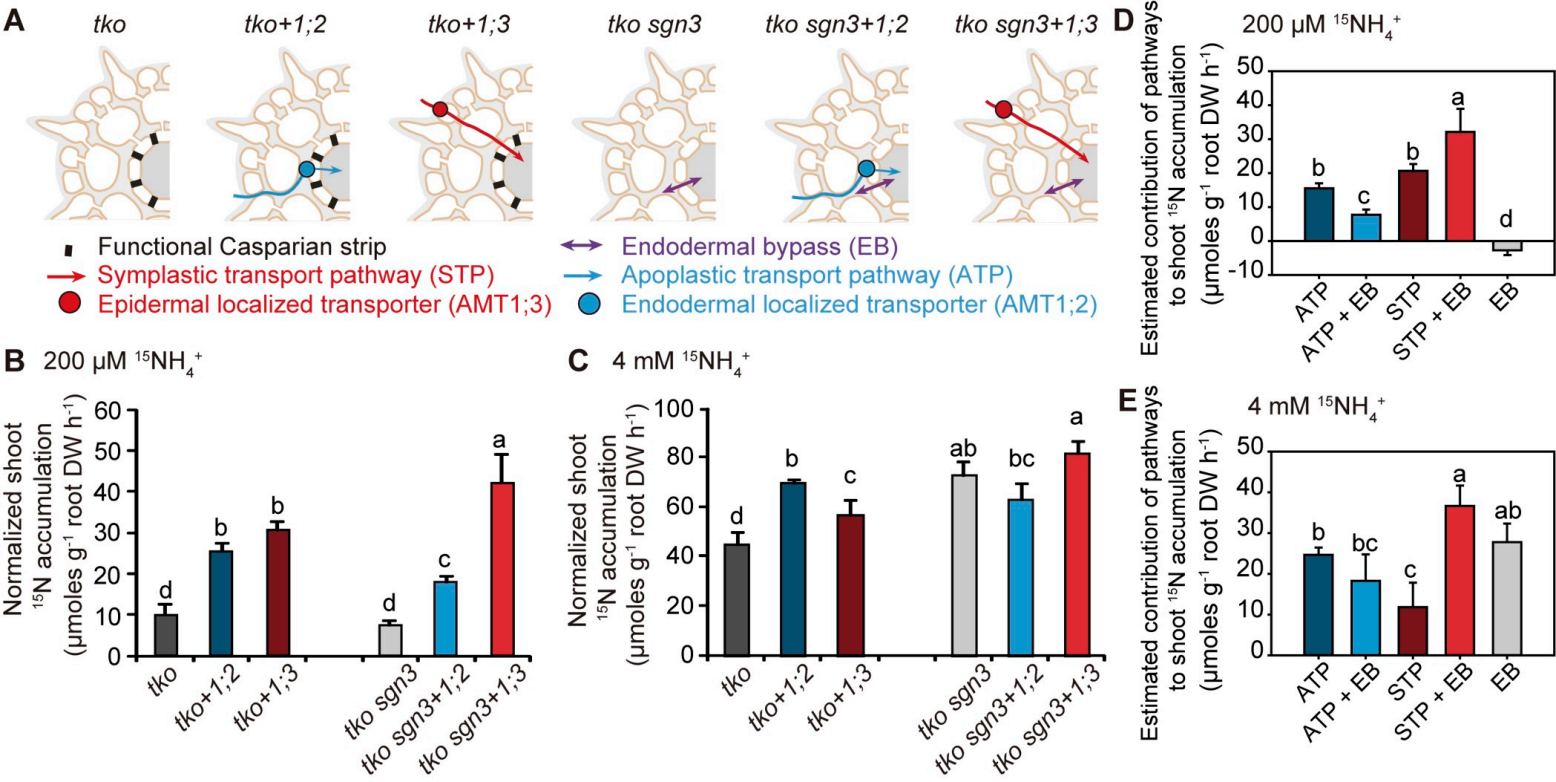
Quantitative contribution of symplastic and apoplastic pathways to radial transport of ammonium in roots. (A) Schematic representation of radial transport pathways in roots of *tko* or *tko sgn3* without or with reconstituted expression of *AMT1;2* (*+1;2*) or *AMT1;3* (*+1;3*). (B and C) Normalized shoot accumulation as readout for radial transport rates of NH_4_^+^-N at 200 μM (B) or 4 mM ^15^NH_4_^+^ (C). (D and E) Estimated contribution of the ATP or the STP in absence or presence of an EB at 200 μM (D) or 4 mM ^15^NH_4_^+^ (E). Values were calculated by subtracting the background of *tko*. Bars represent means ± SD (*n* = 4 biological replicates). Different letters indicate significant differences according to Tukey’s multiple test at *p* < 0.05. Underlying data can be found in S1 Data. ^15^NH_4_^+^, ^15^N-labeled ammonium; AMT, ammonium transporter; ATP, apoplastic transport pathway; DW, dry weight; EB, endodermal bypass; N, nitrogen; *sgn3*, *schengen 3*; STP, symplastic transport pathway; *tko*, *amt1;1 amt1;2 amt1;3*.

To quantify the contribution of individual pathways to radial NH_4_^+^ transport, we introgressed the *sgn3* mutation into the triple *amt1;1 amt1;2 amt1;3* (*tko*) knockout line, which has only 5%–10% of the wild type capacity for high-affinity NH_4_^+^ uptake [12]. The obtained quadruple knockout (*tko sgn3*) had an extended endodermal bypass (Fig 2A) and smaller rosette leaves than *tko* but no visible symptoms of nutrient deficiency (S4 Fig). The presence of such endodermal bypass in a root devoid of the 3 major NH_4_^+^ uptake transporters increased significantly the normalized shoot accumulation of NH_4_^+^-derived N but only at high external supply (*tko* versus *tko sgn3*; Fig 2B and 2C). Although alternative uptake pathways for NH_3_ or NH_4_^+^ likely exist in roots, the absence of a significant difference between wild type and *sgn3* (Fig 1D and 1E) suggests that the increased normalized shoot accumulation of *tko sgn3* relative to *tko* plants at high external ammonium (Fig 2C) is most likely due to the extended endodermal bypass rather than the action of low-affinity transport pathways.

To compare the contribution of the endodermal bypass with that of the ATP or STP, we generated *tko* lines with reconstituted expression of either endodermal *AMT1;2* (*tko+1;2*), thus installing the end point for the ATP at the endodermis, or epidermal *AMT1;3* (*tko+1;3*), thereby establishing an early entry into the STP (Fig 2A). At low external ^15^NH_4_^+^, symplastic transport via AMT1;3 alone conferred slightly higher normalized shoot accumulation for the tracer than the AMT1;2-dependent ATP (Fig 2B and 2D). At the same time, ^15^N accumulation in roots was significantly higher in *tko+1;3* than in *tko+1;2* (S5A and S5B Fig). However, at elevated substrate levels, the difference between *tko+1;3* and *tko+1;2* to *tko* reversed (Fig 2C), and the estimated contribution of the AMT1;2-dependent ATP to shoot ^15^NH_4_^+^ accumulation became twice as high as that through the STP (Fig 2E). Notably, the higher normalized shoot accumulation attributed to the ATP was independent of ^15^N accumulation in roots (S5C and S5D Fig), indicating that root and shoot accumulation of NH_4_^+^-derived N were uncoupled. The radial transport capacity of AMT1;1, AMT1;2, and AMT1;3 together, as reflected by the difference of normalized shoot accumulation between wild type and *tko* (Figs 1D, 1E, 2B and 2C), was somewhat lower than the sum of the 2 individual capacities conferred by AMT1;2 and AMT1;3 (Fig 2D and 2E). This was not unexpected with regard to the fact that AMT1;1 and AMT1;3 can individually compensate for the lacking uptake capacity of each other because the formation of a heterotrimeric complex and concomitant posttranslational down-regulation of interacting monomers was no longer possible [12, 24]. This was also the reason why the contribution of AMT1;1 could be disregarded here.

In conclusion, at low external substrate concentrations, the contribution of the STP to radial transport of NH_4_^+^-N prevails over the ATP, whereas at millimolar NH_4_^+^ supply, the ATP mediated by AMT1;2 confers an approximately 2-fold higher capacity than that of the symplastic route. These results represent, to our knowledge, the first quantitative comparison of individual radial transport pathways for any nutrient.

### Interaction between the endodermal bypass and the symplastic or apoplastic transport pathway

To investigate the cross-talk between radial transport pathways, we hypothesized that the presence of an extended endodermal bypass will decrease radial ^15^NH_4_^+^ transport via the apoplastic or symplastic pathway because tracer reaching the stele may leak out in the absence of a functional CS. Indeed, shoot ^15^NH_4_^+^ accumulation via the AMT1;2-dependent apoplastic pathway in the presence of an endodermal bypass (i.e., *tko sgn3+1;2* versus *tko sgn3*) revealed similar or significantly lower ^15^NH_4_^+^ accumulation relative to the contribution of the ATP alone (Fig 2B and 2C). These results indicated that AMT1;2-mediated ^15^NH_4_^+^ transport across the endodermis was compromised by concomitant NH_4_^+^ efflux through an endodermal bypass. This observation highlights that an intact CS improves the efficiency of the apoplastic transport route by limiting apoplastic backflow out of the vasculature. Unexpectedly, the interaction between an endodermal bypass and the STP was opposite: normalized shoot accumulation of ^15^NH_4_^+^ via AMT1;3 was significantly higher in the *tko sgn3* background (*tko sgn3+1;3* versus *tko sgn3*) than in the *tko* background (*tko +1;3* versus *tko*) (Fig 2B and 2C). Importantly, the estimated contribution of the STP in the presence of an endodermal bypass is higher than either of these transport routes alone, indicating a synergistic interaction between the STP and the endodermal bypass (Fig 2D and 2E). Presumably, part of the NH_4_^+^ transported via the symplastic route was exported into the apoplast during the radial move and profited from unhindered apoplastic diffusion at the endodermis to reach the vascular system. The opposite contribution of AMT1;2 and AMT1;3 in the *tko sgn3* background was not due to altered gene expression, as the expression of *AMT*s was not significantly affected by the *sgn3* mutation (S6 Fig). Moreover, we supplied sufficient amounts of potassium (K) in our hydroponic solution to prevent latent K deficiency in *sgn3* [9]. With this measure, *sgn3* mutants showed no symptoms of nutrient deficiency, and despite slight variations in the accumulation of other mineral elements, all of these were in a usual physiological range and far away from critical deficiency or toxicity levels [25] (S4A and S7 Figs, S1 Table). Thus, our elemental analysis supported that the results with the *sgn3* mutant were largely independent of mineral element disorders or potential interactions between K^+^ and NH_4_^+^ at the level of uptake or xylem loading.

**Fig 3:**
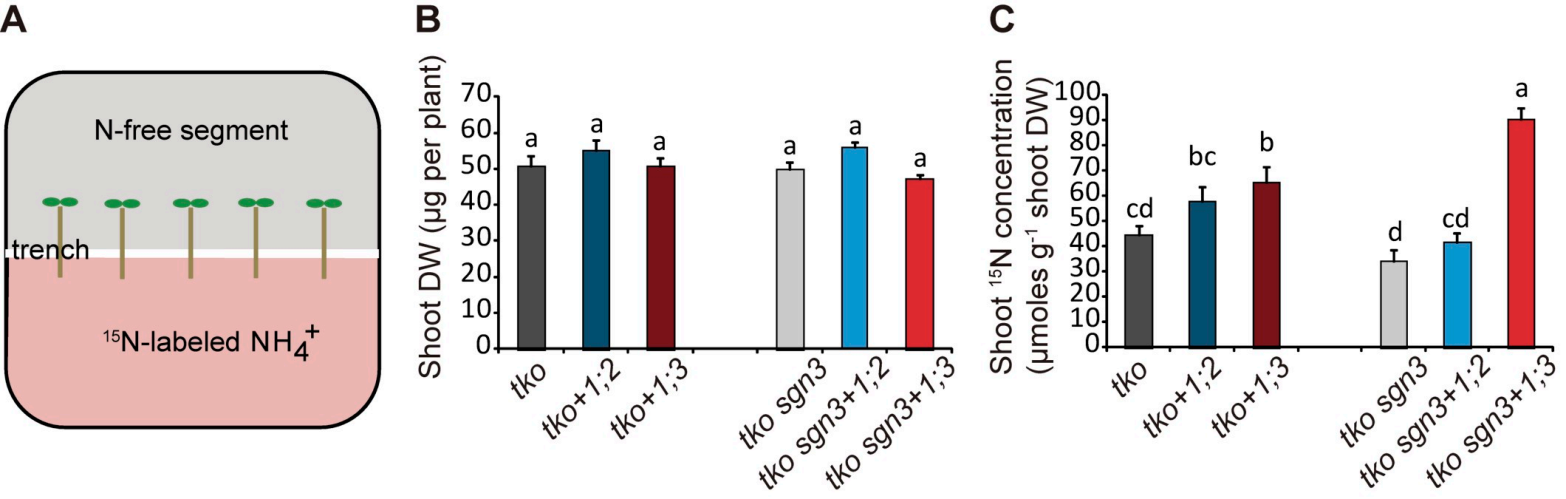
Interaction of the EB with the ATP or STP. (A) Experimental setup for the transport assay on horizontally split agar plates. Plants were placed in a way that only approximately 10 mm of the apical root zone were in contact with the ^15^N-containing segment, while shoots were placed on the N-free upper compartment. (B) Shoot DWs, and (C) ^15^N concentrations in shoots, as readout for radial transport rates. All plants were precultured on one-half MS agar with 1 mM nitrate for 5 d, then on N-free medium for 3 d before transfer to horizontally split agar plates with 4 mM ^15^NH_4_^+^ in the bottom segment. Plants were labeled with ^15^NH_4_^+^ for 6 h. Bars represent means ± SE (*n* = 6–8 biological replicates). Different letters indicate significant differences according to Tukey’s multiple test at *p* < 0.05. Underlying data can be found in S1 Data. ^15^NH_4_^+^, ^15^N-labeled ammonium; ATP, apoplastic transport pathway; DW, dry weight; EB, endodermal bypass; N, nitrogen; one-half MS, half-strength Murashige and Skoog basal salt mixture; STP, symplastic transport pathway.

To verify the opposite interaction between an extended endodermal bypass and the 2 radial transport pathways in an independent growth system, we carried out a transport assay on horizontally split agar plates, in which ^15^NH_4_^+^ was supplemented only to the lower agar segment (Fig 3A). Roots of N-deficient plants were placed across the trench, separating the upper and lower agar compartments so that only approximately 10 mm of the apical root zone were in contact with the ^15^N-containing segment. At this developmental stage, the shoot biomass between *tko* lines and *tko sgn3* lines was almost indistinguishable (Fig 3B). As labeled root segments were too small to measure root dry weights, we determined ^15^N concentrations in shoots as readout for radial transport of ^15^NH_4_^+^-N. Shoot ^15^N concentrations were higher in *tko sgn3+1;3* plants than in *tko+1;3*, thus confirming the synergistic interaction between an endodermal bypass and the STP. On the other hand, we observed a slightly decreased contribution of AMT1;2 in the *tko sgn3* background (*tko sgn3+1;2* versus *tko*+*1;2*; Fig 3C), further supporting that an endodermal bypass antagonizes the ATP.

**Fig 4:**
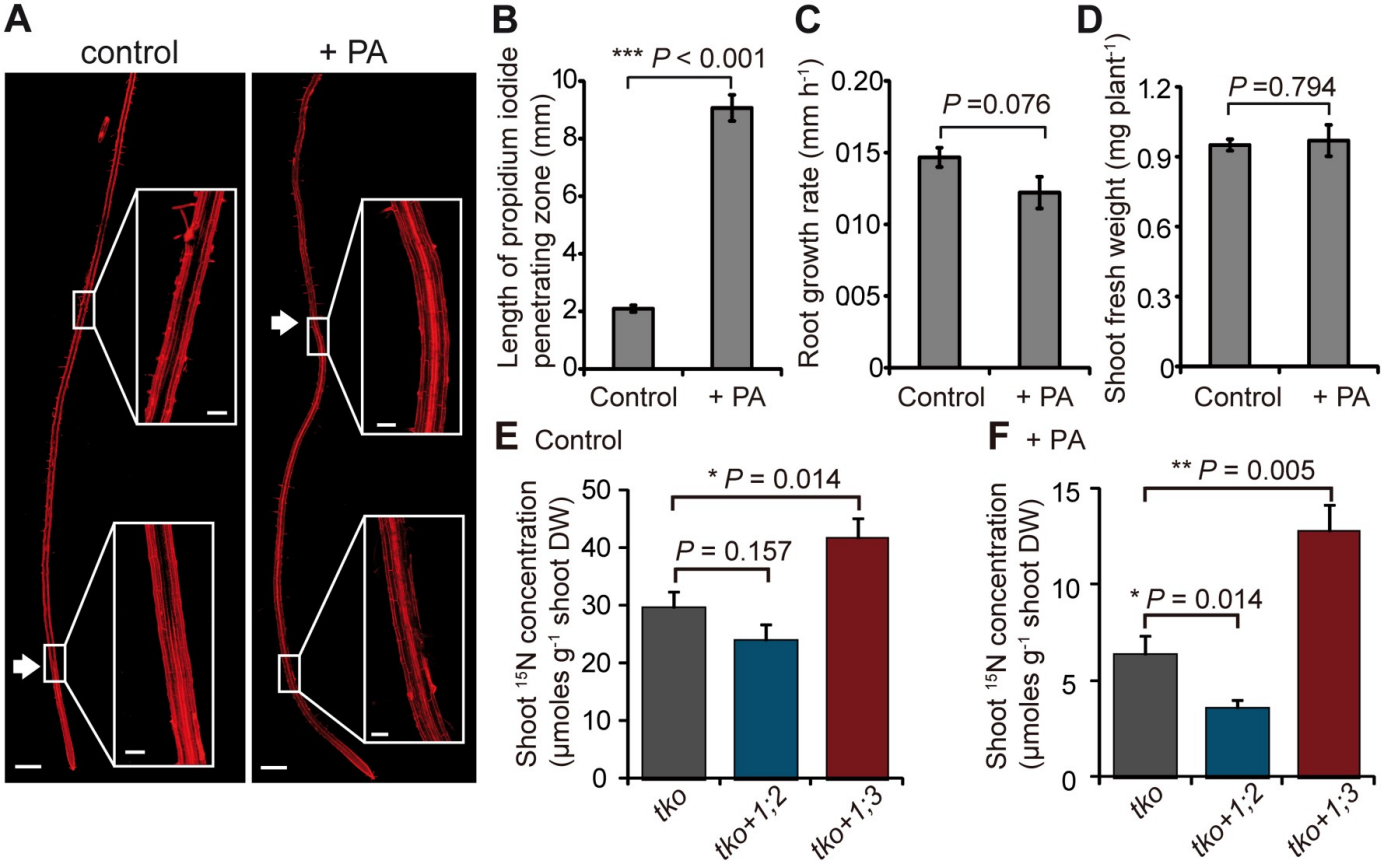
Contribution of the ATP or the STP to radial ammonium transport in the presence of a lignin biosynthesis inhibitor. (A) Tile scans of representative roots, in which the presence of functional CS was verified by PI diffusion. White arrows indicate where PI diffusion was blocked, and inserts highlight PI distribution in selected root zones. Scale bars represent 500 μm in tile scans and 200 μm in inserts. (B) The length of the zone devoid of functional CS was estimated by measuring the length of the PI-penetrating zone. Treatment with PA did not significantly affect root growth rate (C) or shoot fresh weight (D). (E and F) ^15^N concentrations in shoots as readout for radial transport rates. *tko*, *tko+1;2*, and *tko+1;3* plants were either exposed to (E) 0.05% DMSO or (F) 10 μM PA for 48 h before exposure of root segments to 4 mM ^15^NH_4_^+^. Precisely 7 mm of the apical root tips were exposed to ^15^NH_4_^+^, since PA treatment inhibited CS formation up to 9 mm (B). After 6 h, shoots were collected for ^15^N analysis. Bars represent means ± SE. *P* values were calculated using Student *t* test (*n* = 5–10 biological replicates). Underlying data can be found in S1 Data. ^15^NH_4_^+^, ^15^N-labeled ammonium; ATP, apoplastic transport pathway; CS, Casparian strip; N, nitrogen; PA, piperonylic acid; PI, propidium iodide; STP, symplastic transport pathway; *tko*, *amt1;1 amt1;2 amt1;3*.

To further validate our findings independently of the *sgn3* mutation, we treated WT plants with the lignin biosynthesis inhibitor piperonylic acid (PA), which blocks CS formation and hence partially mimics the CS defects obtained by mutating SGN3 [3]. Since the PA effect is confined to newly grown root portions, we carried out the experiment on agar plates and limited the PA treatment to 48 h. Within this period, the zone of unrestricted penetration of the apoplastic tracer propidium iodide (PI) greatly expanded, as the endodermal bypass was now open up to >8 mm from the root tip (Fig 4A and 4B). Notably, this short-term disturbance of CS formation in PA-treated roots did not significantly compromise root growth or shoot biomass formation (Fig 4C and 4D). We then exposed the apical 7 mm of the root tip to ^15^NH_4_^+^ in order to determine the contribution of PA-dependent endodermal bypass. Shoot ^15^N concentrations were strongly suppressed by PA (Fig 4E and 4F), indicating an inhibitory side effect of PA on root physiology. Nevertheless, when compared to *tko*, the endodermal bypass created by PA decreased shoot ^15^N accumulation in *tko+1;2* further but increased shoot ^15^N accumulation in *tko+1;3*, indicating a synergistic interaction (Fig 4E and 4F). Thus, 3 independent approaches provided evidence for the opposite interaction of the endodermal bypass with either the ATP or STP in roots.

### Distinct roles of AMTs in ammonium partitioning between roots and shoots

**Fig 5:**
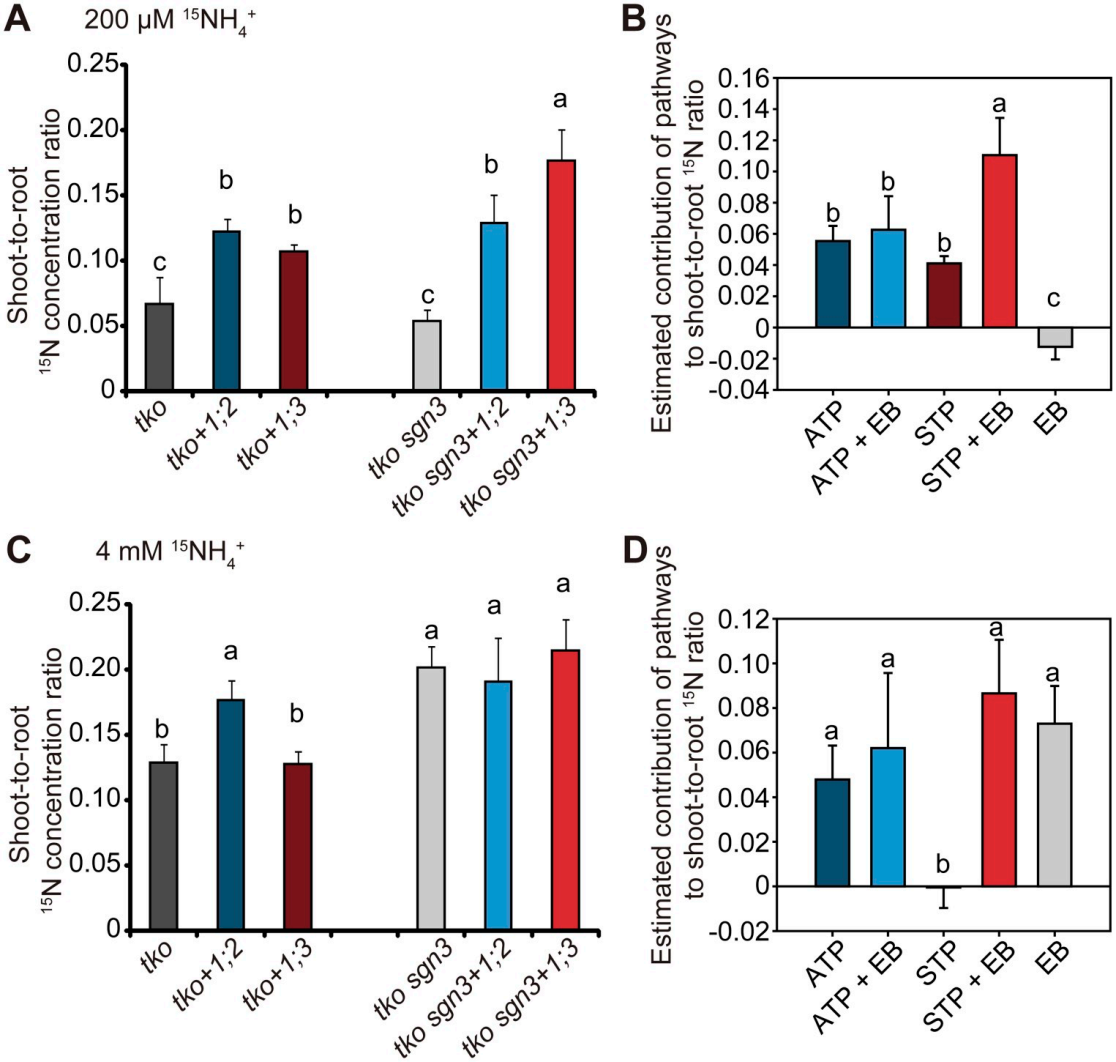
Contribution of the AMT1;2-mediated apoplastic and AMT1;3-mediated symplastic transport pathway to N partitioning between roots and shoots. (A and C) Ratio of ^15^N concentration in shoots to ^15^N concentration in roots of *tko* or *tko sgn3* without or with reconstituted expression of *AMT1;2* (+*1;2*) or *AMT1;3* (+*1;3*). (B and D) Estimated contribution of the ATP or STP in the absence or presence of an EB at 200 μM (B) or 4 mM ^15^NH_4_^+^ (D). Values were calculated by subtracting the background of *tko*. All plants were grown hydroponically for 5 weeks in nutrient solution containing 2 mM nitrate, followed by 3 d of N starvation before transfer to ^15^NH_4_^+^ labeling for 1 h. Shoot-to-root ^15^N concentration ratio was compared when ^15^NH_4_^+^ was supplied at 200 μM (A) or 4 mM concentration (C). Bars represent means ± SD (*n* = 4 biological replicates). Different letters indicate significant differences according to Tukey’s multiple test at *p* < 0.05. Underlying data can be found in S1 Data. AMT, ammonium transporter; ATP, apoplastic transport pathway; EB, endodermal bypass; N, nitrogen; ^15^NH_4_^+^, ^15^N-labeled ammonium; *sgn3*, *schengen 3*; STP, symplastic transport pathway; *tko*, *amt1;1amt1;2amt1;3*.

The overall dominant contribution of the STP to radial nutrient transport raised the question of the biological significance of the ATP. We thus compared root-to-shoot translocation in our mutant lines by calculating shoot-to-root ^15^N concentration ratios. At micromolar NH_4_^+^ supply, AMT1;2 conferred in the *tko* background a slightly higher increase in ^15^N translocation to shoots than AMT1;3, while in the presence of an endodermal bypass, AMT1;3 delivered the most N to shoots, reflecting the synergistic interaction with the endodermal bypass (Fig 5A and 5B). At millimolar supply, shoot N provision profited most from NH_4_^+^ delivered by the AMT1;2-dependent apoplastic pathway or from NH_4_^+^ entering the vasculature via the endodermal bypass (Fig 5C). Without the presence of an endodermal bypass, the AMT1;3-dependent STP alone made no contribution to ^15^N allocation to shoots (Fig 5D). We thus concluded that the AMT1;2-mediated ATP favors partitioning of NH_4_^+^-N to the shoots and contributes to shoot N provision particularly at elevated external supply.

### Relative contribution of individual pathways to radial NH_4_^+^ transport along the root axis

**Fig 6:**
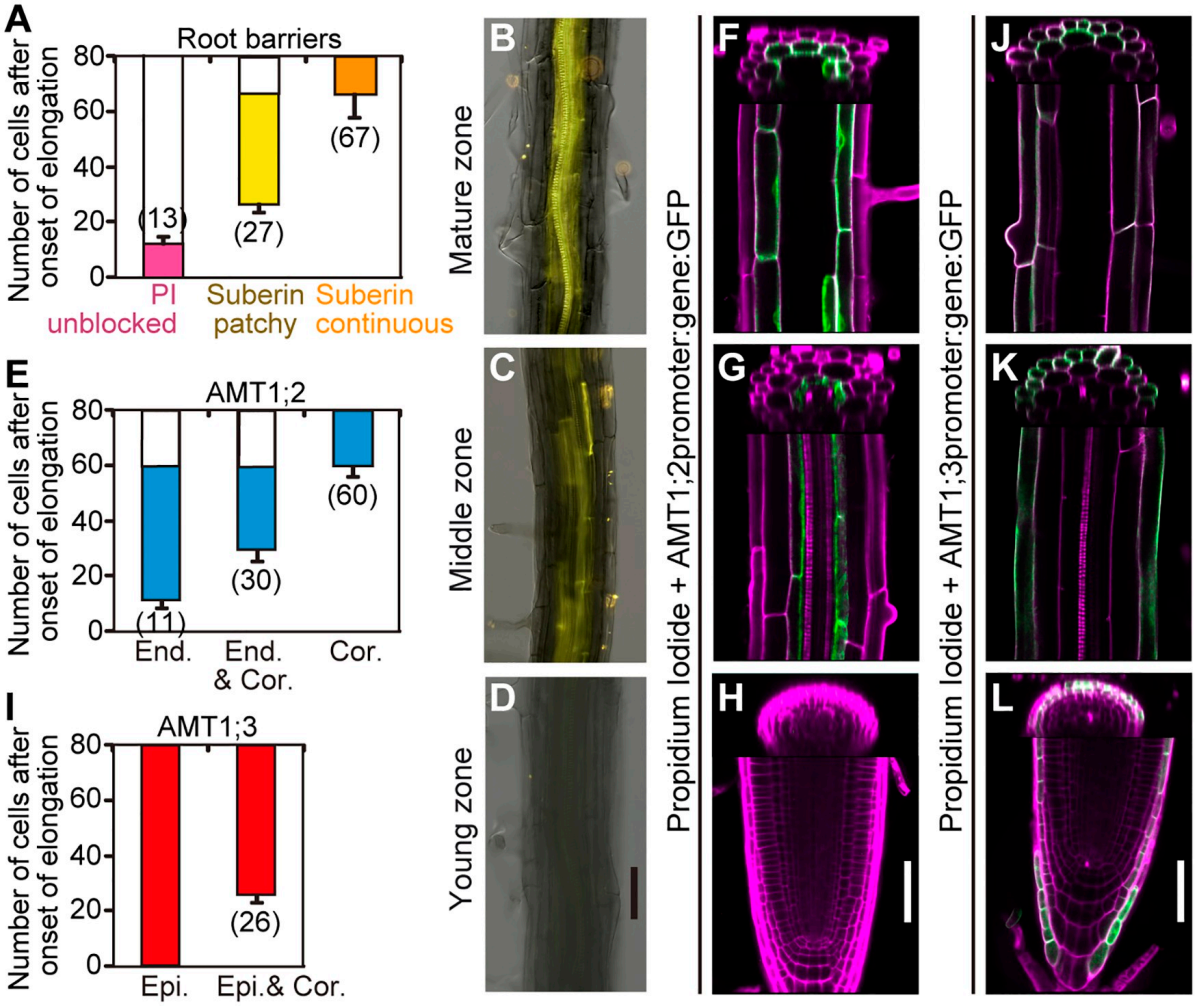
Longitudinal distribution of AMTs and apoplastic diffusion barriers along the root axis. (A) Establishment of functional CSs (PI unblocked), patchy suberin, and continuous suberin along the root axis. CS formation started from the 13th elongated cell onward, indicating that the root zone below the 13th elongated cell down to the initiation of xylem cells represents an endodermal bypass even in WT plants. A zone of patchy suberization extended from the 27th elongated cell up to the 67th cell, from which onward suberin deposition became continuous. (B–D) Fluorol yellow staining shows the presence of suberin in endodermal cells of mature (B), middle (C), and young (D) apical root zones. (E–H) Quantitative assessment (E) and confocal images of root tissue expressing *proAMT1;2*:*AMT1;2*:*GFP* in mature (F), middle (G), and young root zones (H). (I–L) Quantitative assessment (I) and confocal images of root tissue expressing *proAMT1;3*:*AMT1;3*:*GFP* in mature (J), middle (K), and young root zones (L). Roots were stained with PI (magenta). Blockage of PI penetration into the apoplastic space of the stele shows the presence of functional CSs. Overlay of PI and GFP gives white signal. Scale bars in D, H, and L represent 50 μm. In B–D, F–H, and J–L, representative images of >15 plants are shown. In A, E, and I, the data represent means ± SD (*n* ≥ 15 roots). Numbers in brackets are mean values. “Onset of elongation” is defined as the point at which length of an endodermal cell was more than twice its width. Underlying data can be found in S1 Data. AMT, ammonium transporter; Cor., cortex; CS, Casparian strip; End., endodermis; Epi., epidermis; GFP, green fluorescent protein; PI, propidium iodide; WT, wild-type.

Since the occurrence of CSs and suberin lamellae show distinct developmental gradients along the root axis [3,26], we compared their localization with that of the 2 investigated AMTs. The root zone below the 13th elongated cell down to the initiation of xylem cells, which is devoid of a functional CS or suberin deposition, represents an endodermal bypass even in WT plants (Fig 6A and S8 Fig). AMT1;2 was absent from the root tip and present at the endodermis from the 11th to the 60th elongated cell (Fig 6E–6H and S8A–s8D Fig). Notably, from the 30th cell onward, this transporter was also detected in cortical cells, while shifting completely to the cortex from the 60th cell onward. This shift in cell type–specific expression coincided with suberin formation and indicated that the presence of suberin in endodermal cells influenced cell type–specific AMT1;2 localization (Fig 6A–6E and S8A–S8D Fig). By contrast, AMT1;3 expression started from the very root tip in epidermal cells and expanded toward the cortex from the 26th cell onward (Fig 6I–6L and S8E–S8H Fig).

**Fig 7:**
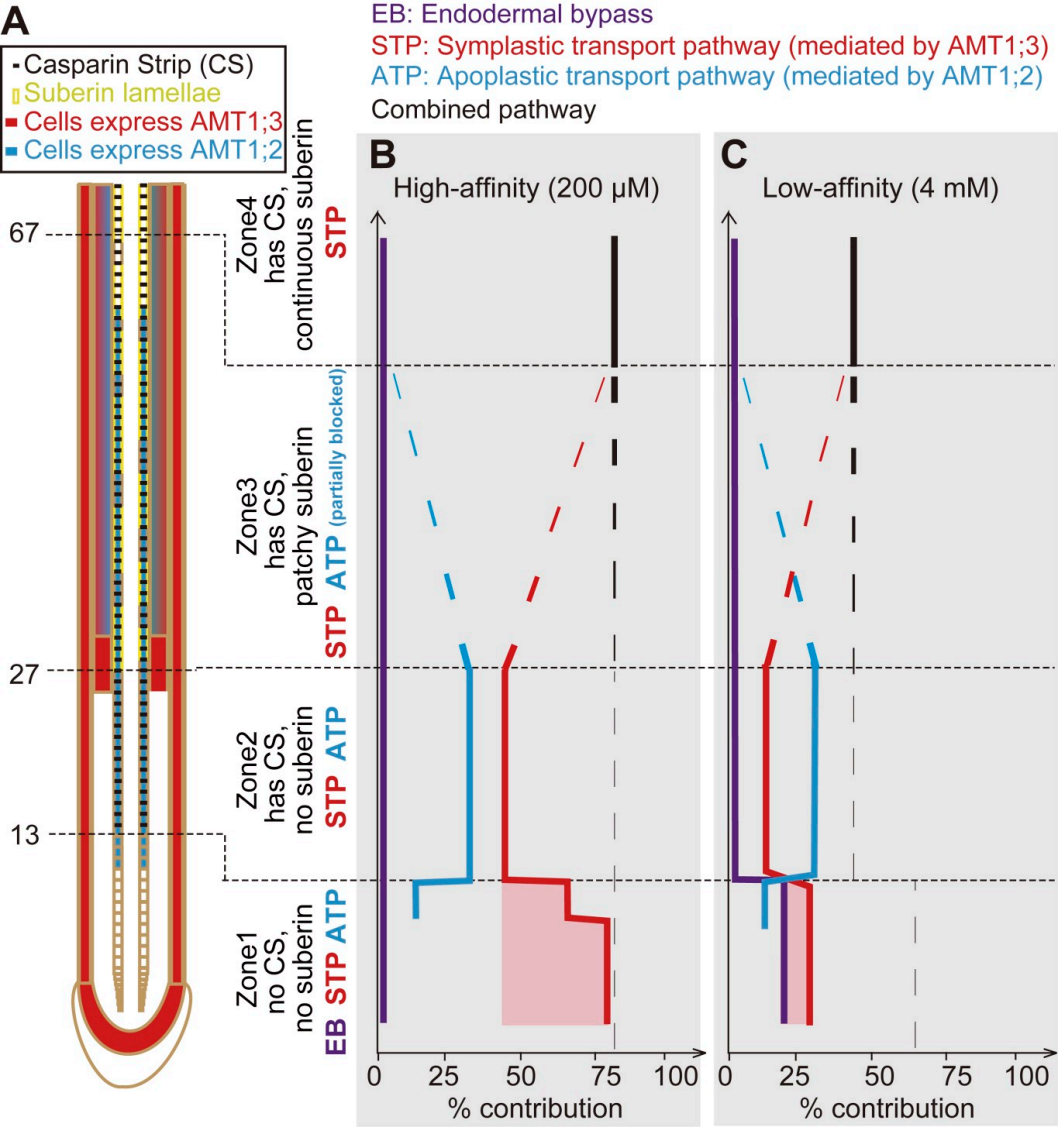
Estimated contribution of the EB, STP, and ATP to radial ammonium movement in different zones along the root of WT plants. (A) Schematic arrangement of apical root zones expressing different combinations of AMT1;2 and AMT1;3 and apoplastic barriers. (B and C) Relative contribution of the EB as well as the ATP and STP in the high-affinity (B) or low-affinity range (C). The root axis is divided into 4 zones based on longitudinal gradients of CSs and patchy or continuous suberin. Calculations on the relative contribution of individual pathways are based on radial transport rates measured in hydroponic experiments (Fig 2 and S2 Table). The red shaded area indicates synergistic action between the EB and STP. Underlying data can be found in S1 Data. AMT, ammonium transporter; ATP, apoplastic transport pathway; CS, Casparian strip; EB, endodermal bypass; STP, symplastic transport pathway; WT, wild-type.

Regarding the establishment of functional radial transport pathways, the localization pattern of the 2 NH_4_^+^ transporters and the apoplastic barriers allowed differentiating 4 zones along the axis of an Arabidopsis root (Fig 7A). We then projected our estimates for the contribution of the individual radial pathways along the longitudinal axis of a WT root to build a model that estimates the relative contribution of different transport pathways to radial NH_4_^+^ transport in roots. In the high-affinity range, for which up to 80% of radial NH_4_^+^ transport depends on AMTs, the EB does not contribute to radial NH_4_^+^ transport along the whole root axis (Figs 2B and 7B, and S2 Table). In “zone 1,” radial transport is completely dominated by the AMT1;3-dependent STP, which profits from the synergistic action of the EB. Once AMT1;2 is expressed, its contribution is rather modest (approximately 16%), as it is negatively affected by the presence of an EB. When the CS becomes established (zone 2), the STP and ATP contribute by 45% and 34%, respectively (Figs 2B and 7B, and S2 Table). With progressing suberization of the endodermis (zone 3 + zone 4), the estimated contribution of the ATP ceases and is taken over by a combined pathway, which is defined by the expression of both transporters in cortical cells mediating uptake of apoplastically transported NH_4_^+^ across the epidermal layer into the symplastic route.

In the low-affinity range, the overall contribution of the AMT- and *sgn3*-dependent pathways is only 45%–65%, as NH_4_^+^ is transported additionally by not yet fully characterized low-affinity transporters (Figs 2C and 7C, and S2 Table). In this case, the endodermal bypass contributes by 22% to radial NH_4_^+^ transport in “zone 1.” AMT1;3-mediated symplastic transport, in turn, contributes by 29%, albeit in a synergistic manner, with the endodermal bypass. In contrast, the contribution of AMT1;2 is strongly decreased by the endodermal bypass (Fig 2C). In “zone 2,” AMT1;2-dependent apoplastic transport dominates radial NH_4_^+^ transport, with 30% over the 15% of AMT1;3-dependent symplastic transport. In “zone 3,” the contribution of the ATP decreases due to suberization of endodermal cells, and in “zone 4,” the contribution was taken over by a combined pathway.

**Fig 8:**
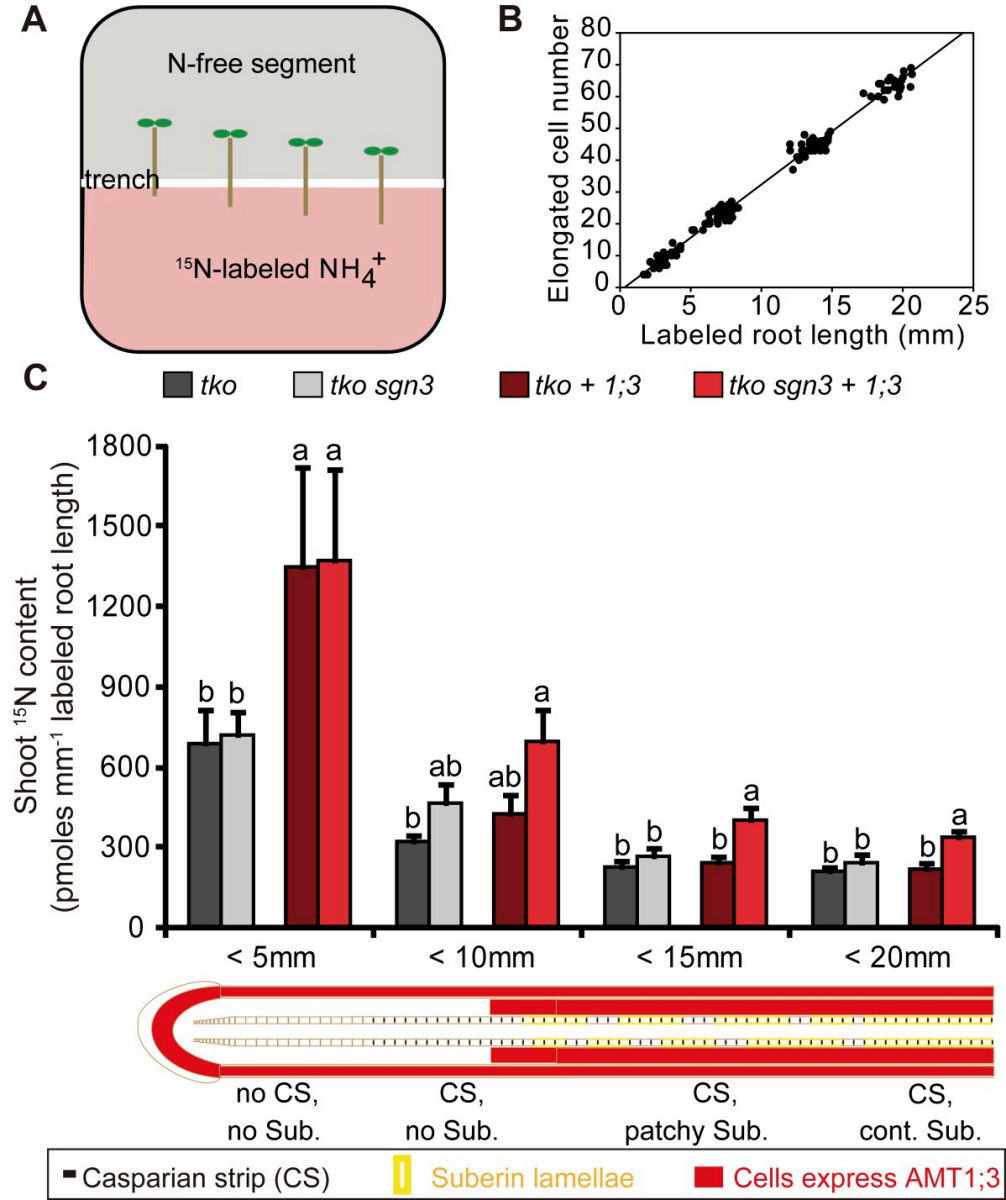
Interaction between the EB and the STP in a root zone–dependent ^15^N-labeling assay. (A) Experimental design for the assessment of root zone–dependent radial transport pathways. Plants were placed in a way that root segments of different lengths were in contact with ^15^NH_4_^+^, while shoots were placed on the N-free upper compartment. (B) Correlation between “labeled root length” and “labeled elongated cell number.” (C) ^15^N contents in shoots resulting from exposure of indicated root segments to ^15^NH_4_^+^. All plants were precultured on one-half MS agar with 1 mM nitrate for 5 d, then on N-free medium for 3 d before transfer to horizontally split agar plates with 4 mM ^15^NH_4_^+^ in the bottom segment. Values were normalized to labeled root length. Bars represent means ± SE (*n* = 6–9 biological replicates). Significant differences are indicated by different letters at *p* < 0.05 according to Tukey’s test. Underlying data can be found in S1 Data. EB, endodermal bypass; N, nitrogen; ^15^NH_4_^+^, ^15^N-labeled ammonium, one-half MS, half-strength Murashige and Skoog basal salt mixture; STP, symplastic transport pathway.

To validate our model, we exposed apical root tips of different length to a ^15^NH_4_^+^-containing agar plate compartment and correlated root lengths touching the tracer with counted elongated cell numbers (Fig 8A and 8B). Samples were grouped into 4 classes, corresponding to the 4 zones with distinct combinations of transport pathways along the root axis (Fig 7A). With this procedure, radial transport of ^15^NH_4_^+^ was integrated over longitudinal root segments with different sets of radial transport pathways. Longitudinal gradients of CS or suberin were neither affected by the *sgn3* mutation nor by AMT1;3 expression (S9 Fig). Shoot ^15^N accumulation after 4 h of labeling was related to root length and taken as readout for the radial transport rate of each root zone (Fig 8C). In all lines, the highest radial transport rate was detected in the most apical part. The contribution of the STP (*tko+1;3* versus *tko*) to radial transport, here represented by shoot accumulation of ^15^NH_4_^+^, was particularly significant in “zone 1” (<5 mm), where CS are not yet formed (Figs 7A and 8C). The endodermal bypass alone (*tko sgn3* versus *tko*) had no significant impact on shoot ^15^N accumulation. The comparison between *tko+1;3* and *tko sgn3+1;3* showed that loss of a functional CS increased AMT1;3-mediated symplastic transport capacity, especially in “zone 3” and “zone 4” (Fig 8C), further indicating a synergistic effect between the STP and the EB.

### Discussion

While the contribution of the ATP and STP to radial transport of nutrients through the root tissue has been adopted as a general principle in classical textbooks [6,7], the quantitative share between these 2 pathways for radial transport of any nutrient has remained elusive. Here, we took advantage of the differential cell type–specific expression of AMT1–type transporters in combination with the CS-defective mutant *sgn3* to dissect radial transport pathways for NH_4_^+^ and to determine their quantitative contribution. Thereby, we discovered previously unanticipated interactions between radial transport pathways and differential roles of ATP and STP for nutrient partitioning between roots and shoots.

A prerequisite for the dissection of radial transport pathways is the availability of membrane transport proteins mediating either early substrate passage via the epidermis into the symplastic continuum or completing the apoplastic transport by transmembrane passage into endodermal cells. Unlike for most other nutrients, such transporters were known for NH_4_^+^ and previously characterized for their contribution to root uptake [12,16]. Assessing their contribution to radial NH_4_^+^ transport required a longer period of exposure to the tracer and revealed a higher capacity of the AMT1;3-mediated symplastic route at low external NH_4_^+^ supply but a higher capacity of the AMT1;2-mediated apoplastic route at elevated supply (Fig 2B–2E). Although AMT1;2 is characterized by lower substrate affinity, i.e., 234 μM relative to 61 μM for AMT1;3 [12], better adapted biochemical transport properties to higher apoplastic NH_4_^+^ concentrations alone cannot explain its superior contribution to shoot NH_4_^+^ accumulation (Fig 5). AMT1;2-dependent NH_4_^+^ transfer to shoots was uncoupled from NH_4_^+^ accumulation in roots (Fig 2E and S5D Fig) because apoplastically transported NH_4_^+^ circumvents retention by root cells in favor of direct movement to the stele. Thus, the ATP may function as a “fast track” for nutrient delivery to the shoot. The reason for its greater importance at elevated external substrate supplies may lie in a lower number of low-affinity NH_4_^+^ transporters competing with AMT1;2 for NH_4_^+^ transport across the endodermis, whereas low-affinity NH_4_^+^ transporters in epidermal cells may be more abundant, masking the contribution of AMT1;3. At least this holds true for AMT2;1, which has been shown to localize in the epidermis and cortex of N-deficient roots, where it confers approximately 10%–25% of the low-affinity uptake capacity [13]. A supposedly differential contribution of these poorly characterized low-affinity transport systems for NH_4_^+^ in different cell types may restrict direct comparison of AMT1;2- versus AMT1;3-mediated radial transport capacities. In the high-affinity range, >90% of the NH_4_^+^ uptake capacity in roots relies on AMT1;1, AMT1;2, and AMT1;3 [12]. Respecting the compensatory increase in AMT1;3 capacity in the absence of AMT1;1 [12,24], quantitative estimates of the share between apoplastic and symplastic pathways to radial transport in the high-affinity range are unlikely to be affected by other transport systems.

It is important to note that the contribution of an extended endodermal bypass might change according to growth conditions, particularly to substrate concentration, root pressure, or transpiration rates. Therefore, we assessed ^15^N shoot accumulation during the light phase, when transpiration is high and root pressure is low, thereby suppressing the effect of root pressure on xylem transport rates. Such conditions allowed investigating the contribution of AMT1;2- and AMT1;3-dependent pathways in presence or absence of an extended EB more directly and at a higher resolution. In our experimental conditions, the ATP reached highest capacity when CSs were intact, and this capacity was as high as that mediated by the EB alone (Fig 2C). We therefore conclude that the membrane transport steps required for xylem loading, i.e., NH_4_^+^ transfer to pericycle cells and subsequent export to xylem vessels, are not limiting for root radial transport. Hence, a quantitative comparison between the ATP and STP for NH_4_^+^, as addressed by our approach, appears valid.

Apart from determining the contribution of individual pathways to radial NH_4_^+^ transport, we also focused on their interplay. This question is of particular biological relevance due to the longitudinal gradients of apoplastic barrier formation, which generate a suite of possible radial pathway interactions. At the primary root axis, an interplay between all 3 pathways is confined to a small zone, in which epidermal AMT1;3 is coexpressed with endodermal AMT1;2, while CS or suberin are not yet formed (Figs 6 and 7). In the *sgn3* mutant, the largely expanded zone with an endodermal bypass enhanced radial transport, as shown by higher normalized shoot accumulation of the apoplastic tracer Sr^2+^ (Fig 1B and 1C), whereas EB of NH_4_^+^ was outcompeted by AMT-mediated radial transport pathways (Figs 1D–1E and 2B–2C). Surprisingly, we found that the EB has an opposing role on the 2 radial transport pathways, which we confirmed in 3 independent methodological approaches, i.e., by a) examining *tko* and *tko sgn3* lines with reconstituted expression of AMT1;2 or AMT1;3 in 5-week-old hydroponically grown plants (Fig 2), b) in 8-d-old agar-grown plants (Fig 3), or c) by circumventing the use of the *sgn3* background with the help of a lignin biosynthesis inhibitor (Fig 4). A compromising action of the EB on the ATP occurs when NH_4_^+^ leaking out through a defective CS must be retrieved by AMT1;2, i.e., by the same transporter that is already engaged in the apoplastic transport route. Although the net contribution of an open EB was 0 at low external NH_4_^+^ supply, it promoted shoot ^15^N accumulation via the AMT1;3-dependent symplastic route (Fig 2D). Presumably, part of NH_4_^+^ transported via the symplastic route was exported into the apoplast during the radial move and reached the vascular system by profiting from unhindered apoplastic diffusion through the endodermal cell layer. NH_4_^+^ efflux has been extensively characterized in physiological studies and shown to be dominated by the export of NH_3_ [27, 28]. Currently, it is not clear which membrane transporters mediate NH_3_ export from root cells, but export across the plasma membrane may be facilitated through NH_3_-transporting aquaporins, as shown for tonoplast intrinsic proteins [29] or through AMT2;1, which alters its localization toward inner root cells under NH_4_^+^ nutrition [13] and is able to permeate NH_3_ [30]. Such synergistic interplay between the STP and the EB may be regarded as experimental evidence for the so-called coupled transcellular transport pathway [1]. This third transport pathway has been postulated to couple repeated steps of symplastic and apoplastic transport based on the polarized localization of importers and exporters, as shown for silicon or boron [31–33].

The relative contribution of the individual radial pathways as well as their interplay is subject to root zonation and the formation of apoplastic barriers. As radial NH_4_^+^ transport via the AMT1;2-mediated ATP was decreased by an extended endodermal bypass in the most apical root zone and progressively sealed by suberin deposition in a shootward direction, an efficient ATP was restricted to a zone between 13 and approximately 67 cells above the first elongated root cell (Figs 6 and 7). Thus, the “fast track” for NH_4_^+^ delivery to the shoot is already in place as soon as the primary root explores new nutrient-rich soil layers and establishes an almost equal share of NH_4_^+^ partitioning between the shoot and the roots, since NH_4_^+^ provision to roots depends mainly on the STP (Fig 5). Since elevated substrate supply can push the relative share of NH_4_^+^ in favor of the shoot, apoplastic NH_4_^+^ transport may finally contribute to enhanced shoot growth under ample N supply. From a general perspective, this principle appears advantageous during root foraging, when soil nutrients are vertically translocated and accumulate in deeper soil layers [34]. However, it is noteworthy that the apoplastic transport route is highly sensitive to environmental conditions. Abiotic stress factors, including high salt or K deficiency, accelerate the suberization of endodermal cells via abscisic acid (ABA) and can restrict the zone of substrate-accessible endodermal transporters to less than 50% [35]. This may also be the reason why the suberin-enriched mutants *esb1* and *myb36* showed lower normalized shoot accumulation of ^15^NH_4_^+^ (S3 Fig). Endodermal transporters directly impact the nutrient composition of the shoot and, in turn, may be subject to straight control by the nutritional status of the shoot. Thus, characterizing transport processes across the endodermal plasma membrane in both directions and quantifying the contribution of the ATP versus the STP is highly relevant. Such knowledge might help specify breeding targets in plants, enabling more selective nutrient translocation to shoots, e.g., to meet the increasing N demand of shoots growing under elevated carbon dioxide (CO_2_), decreasing shoot accumulation of xenobiotics, or fortifying seeds with essential mineral elements.

## Materials and methods

### Generation of multiple mutant lines

*tko* plants (*amt1;1amt1;2amt1;3*) were obtained from a selfed F2 population after backcrossing a homozygous *amt1;1-1amt1;3-1amt2;1-1amt1;2–1* quadruple insertion line [12] to WT Col-0. *tko* and *sgn3;3* (in Col-0 background, SALK_043282) [9] were crossed and selfed to obtain an F2 population. Within the F2 population, homozygous lines of *tko+AMT1;2* (*tko+1;2*), *tko+AMT1;3* (*tko+1;3*), *tko sgn3*, *tko sgn3+AMT1;2* (*tko sgn3+1;2*), and *tko sgn3+AMT1;3* (*tko sgn3+1;3*) were selected by PCR. For details of primers for genotyping, see S3 Table. Homozygous *tko* and *sgn3;3* from this population served as reference lines. All homozygous mutant lines were selfed, and seeds from F3 generations were used in all experiments. In this work, 1 biological replicate represents a sample consisting of 2 plants from the same line or treatment (except for xylem sap, for which 4 plants were combined as 1 sample). The number of biological replicates indicated in the figure legends represents the number of independent biological replicates (originating from same line/treatment but different pot or agar plate).

### Measurement of normalized shoot accumulation rates in *Arabidopsis thaliana*

Plants were grown hydroponically for 5 weeks, as previously described [12,16]. Nitrate was used as the sole N source for preculture in all experiments. The climate chamber had a day–night regime of 10 h (22 °C)/14 h (18 °C), 200 μmol m^−2^ s^−1^ light intensity, and 70% humidity. Prior to labeling, plants were precultured for 3 d on an N-free nutrient solution. Just before ^15^N or Sr^2+^ labeling, plant roots were rinsed in 1 mM CaSO_4_ for 1 min to remove nutrients from the apoplast and then transferred to the N-free solution containing different concentrations of (^15^NH_4_)_2_SO_4_ (98 atom% ^15^N) or SrCl_2_. After 1 h, roots were washed again in 1 mM CaSO_4_ for 1 min to remove tracers from the apoplast. Shoots and roots were harvested, freeze dried, and ground to fine powder. To harvest xylem sap samples, plants were decapitated below the rosette, and xylem sap from 4 plants was combined in 1 sample. ^15^N concentrations were determined by isotope-ratio mass spectrometry (DELTAplus XP, Thermo-Finnigan). Other mineral elements were determined by high-resolution inductively coupled plasma mass spectrometry (Element 2, Thermo Fisher Scientific). Sr was determined by high-resolution inductively coupled plasma mass spectrometry (ICP-MS, Element 2, Thermo Fisher Scientific). Normalized shoot accumulation was calculated by normalizing total Sr^2+^ or ^15^N shoot content to root dry weight and labeling time. The formula used is as follows: ^15^N accumulation rate in shoot (or xylem exudate) = (sample ^15^N abundance − natural ^15^N abundance) × total N concentration × shoot (or xylem exudate) dry weight/(^15^N molecular weight × ^15^N purity × labeled time × root dry weight). Natural ^15^N abundance was obtained by measuring unlabeled leaf material. For harvest, 2 plants were taken as 1 sample in 4 replicates for each data point. Normalized shoot accumulation in different lines was compared in 3 independent experiments.

### Microscopy, histology, and quantitative analysis

Construction of *promoterAMT1;2*:*ORF*:*GFP* and *promoterAMT1;3*:*ORF*:*GFP* lines were described previously [12,16]. Plants were precultured on a one-half Murashige and Skoog (MS) medium containing 1 mM NO_3_^-^ as the sole N source, 2.5 mM 2-(N-morpholino) ethanesulfonic acid (MES) (pH 5.7), 1% sucrose, and 0.8% Difco agar (Becton Dickinson). Five-d-old seedlings were transferred to the N-free one-half MS medium for 3 d. N-deficient plants were used for visualization of GFP and root barriers. For visualizing propidium iodide (PI) penetration, plants were stained with PI (10 μg mL^-1^) for 10 min in the dark [3,36]. PI-dependent red fluorescence and AMT:GFP–dependent green fluorescence in different root zones were observed with a confocal laser scanning microscope Zeiss LSM 780 (Carl Zeiss). Excitation/emission wavelengths of 488 nm/490–540 nm and 561 nm/650–710 nm were used for detection of GFP signals and PI signals, respectively. Cross sections from each zone were reconstructed from z-stacks of longitudinal confocal sections. For visualizing suberin, plants were stained with Fluorol Yellow 088, as described previously [3], and observed with a conventional light microscope (Zeiss Axio Imager M2, Carl Zeiss) using a GFP filter. For quantification, “onset of elongation” was defined as the zone where an endodermal cell length was more than twice its width [3]. More than 20 plants were counted. Experiments were repeated at least 3 times.

### ^15^N-labeling assay for root zone–dependent radial transport

Arabidopsis seeds were surface sterilized and precultured on one-half MS medium containing 1 mM NO_3_^-^ as the sole N source, 2.5 mM MES (pH 5.7), 1% sucrose, and 0.8% Difco agar (Becton Dickinson). Five-d-old seedlings were transferred to one-half MS medium without N and supplemented with 2.5 mM MES (pH 5.7), 1% sucrose, and 0.8% Difco agar. After 3 d, plants with similar root size were transferred to horizontally split agar plates containing the ^15^N tracer in the bottom segment. To prepare the ^15^N-labeled agar plates, 50 ml one-half MS medium without N, supplemented with 2.5 mM MES (pH 5.7) and 1% Difco agar, was spread on the agar in a 12-cm square Petri dish. No sucrose was added in order to slow down the root growth. A trench of 5-mm width was cut in the solidified agar to avoid ^15^N diffusion between the upper and lower agar segments. (^15^NH_4_)_2_SO_4_ (98 atom% ^15^N) stock solution was spread on the lower agar segment to reach a final ^15^NH_4_^+^ concentration of 4 mM. Petri dishes were placed in vertical orientation in a growth cabinet at a day–night regime of 10 h (22 °C)/14 h (19 °C) and a light intensity of 120 μmol m^-2^ s^-1^ during the day period. In each treatment, 10 plants were placed on the agar plate in a way that approximately 2 mm, 5 mm, 10 mm, or 15 mm of the apical primary root zone touched the ^15^N-containing bottom agar segment, while the shoot was placed on the upper segment (-N agar). Eight-d-old seedlings were used for this ^15^N transport assay to avoid interference from lateral roots and to make sure that only a predefined root portion was in contact with the tracer. For each line, 30 plants were placed on 3 independent plates to normalize the variation on different agar plates. After 4 h of incubation, shoots were separated at the hypocotyl and dried separately in tin capsules. To measure the shoot ^15^N concentration in each plant, whole shoots (about 40 μg dry weight) were mixed with BBOT isotope reference standard (atom percent: 0.366, N percent: 6.5, HEKAtech GmbH) in order to reach the detection limit for total N. Shoot ^15^N concentration was determined by isotope-ratio mass spectrometry (DELTAplus XP, Thermo-Finnigan). Labeled root segments were cut off, mounted on slides, and scanned by an Epson Expression 10000XL scanner (Seiko, Epson). Root lengths were determined by Smartroot software V4.126, and the number of cells from the start of cell elongation onward was counted under differential interference contrast using a conventional light microscope (Zeiss Axio Imager M2, Carl Zeiss). Since the roots grew nearly 2 mm during this 4-h period, final labeled root length was grouped into classes of <5 mm, <10 mm, <15 mm, and <20 mm. For each root length class, 6–9 plants from 3 agar plates were analyzed to yield 1 data point.

### Lignin inhibitor assay

Col-0 plants were precultured on one-half MS agar with 1 mM NO_3_^-^ for 5 d and then transferred for 3 d to agar plates without N. During the last 48 h of growth on the N-free medium, part of the plants were transferred to the same medium containing either 0.05% DMSO (control) or 10 μM of the lignin biosynthesis inhibitor piperonylic acid (PA). To verify the presence of functional CS, roots were stained with PI (10 μg mL^-1^) for 10 min in the dark. For shoot ^15^N analysis, *tko*, *tko+1;2*, and *tko+1;3* plants were precultured as described above and then either exposed to 0.05% DMSO or 10 μM PA for 48 h before transferring to horizontally split agar plates. Since PA treatment inhibited CS formation up to 9 mm, precisely 7 mm of the apical root tips were exposed to 4 mM ^15^NH_4_^+^. After 6 h, shoots were collected for ^15^N analysis, as described above.

### Real-time quantitative PCR

Total RNA was extracted using the QIAzol Lysis reagent (Qiagen) following the manufacturer’s instructions. Prior to cDNA synthesis, samples were treated with DNase (Thermo Fisher Scientific). Reverse transcription was performed using SuperScript II (Thermo Fisher Scientific) reverse transcriptase and Oligo(dT)12-18. Real-time PCR was performed using a Mastercycler ep realplex (Eppendorf) and QuantiTect SYBR Green qPCR mix (Qiagen) using the primers listed in S3 Table. Relative transcript levels were calculated according to Vandesompele and colleagues (2002) [37].

## Supporting information

S1 Fig Determination of radial transport rates in roots. (A) Schematic representation of radial nutrient movement across the root and contribution of root pressure and transpiration to nutrient translocation to the shoot. (B) ^15^N accumulation rates in roots, shoots, and xylem sap of WT plants supplied with 200 μM ^15^NH_4_^+^. The data represent means ± SD (*n* = 4 biological replicates). Plants were grown hydroponically for 6 weeks in full nutrient solution followed by 0–3 d of N starvation to stepwise induce *AMT1* transporters. ^15^N accumulation in roots from intact (dashed line, blue triangles) or decapitated (dashed line, orange triangle) plants increased steadily due to elevated expression of *AMT*s. Irrespective of whether radial transport rates were determined on the basis of ^15^N accumulation in the shoot (solid lines, blue squares) or in the xylem sap (solid lines, orange squares), radial transport rates leveled off, reflecting saturated xylem loading capacities. Relative to shoot ^15^N, xylem sap contained only one-third ^15^N, which was mostly likely due to the lack of transpiration as an additional driving force for root-to-shoot translocation of ^15^N. To properly reflect in planta conditions, ^15^N accumulation in shoots was considered as readout for root radial transport. Underlying data can be found in S1 Data. AMT, ammonium transporter; Cor., cortex; DW, dry weight; End., endodermis; Epi., epidermis; ^15^NH_4_^+^, ^15^N-labeled ammonium; N, nitrogen; Per. Pericycle; WT, wild-type; Xyl., xylem.
(TIF)

S2 Fig Role of the endodermal bypass for Sr^2+^ and NH4+ accumulation in roots. Accumulation of Sr^2+^ (A, B) or ^15^NH_4_^+^ (C, D) in roots of WT (Columbia-0) and *sgn3* plants when supplied at an external concentration of 200 μM or 4 mM. Plants were grown under the same conditions as in Fig 1. Bars represent means ± SD. *P* values were calculated using Student *t* test (*n* = 4 biological replicates). Underlying data can be found in S1 Data. ^15^NH_4_^+^, ^15^N-labeled ammonium; *sgn3*, *schengen 3*; Sr^2+^, strontium ion; WT, wild-type.
(TIF)

S3 Fig Ammonium accumulation rates in different mutants with defects in apoplastic barrier formation. Shoot or root dry weights (A and C) and shoot or root accumulation (B and D) of ^15^NH_4_^+^ in WT (Columbia-0), *sgn3*, *esb1*, and *myb36* mutant plants. Plants were exposed to 200 μM (A and B) or 4 mM (C and D) ^15^NH_4_^+^. Bars represent means ± SD (*n* = 4 biological replicates). Different letters indicate significant differences according to Tukey’s multiple test at *p* < 0.05. Underlying data can be found in S1 Data. ^15^NH_4_^+^, ^15^N-labeled ammonium; *esb1*, *enhanced suberin 1*; *myb36*, *myb domain protein 36*; *sgn3*, *schengen 3*; WT, wild-type.
(TIF)

S4 Fig Shoot growth phenotype and presence of functional CSs in mutant lines. (A) Shoot growth of *tko*, *tko+1;2*, *tko+1;3*, *tko sgn3*, *tko sgn3+1;2*, and *tko sgn3+1;3* mutant lines. All plants were grown hydroponically for 5 weeks in nutrient solution containing 2 mM NO_3_^-^, followed by 3 d of N starvation. Scale bars = 1 cm. The experiment was repeated 3 times, and representative images are shown. (B) Establishment of functional CSs was measured by counting at which elongated cell number the penetration of PI into the apoplastic space of the stele was blocked. Roots from 8-d-old agar-grown plants were stained with PI for 10 min. Bars represent means ± SD. At least 10 roots per mutant line were assessed. Continuous bars indicate that PI was not blocked up to >40 cells. Underlying data can be found in S1 Data. CS, Casparian strip; N, nitrogen; NO_3_^-^, nitrate; PI, propidium iodide; *sgn3*, *schengen 3*; *tko*, *amt1;1amt1;2amt1;3*.
(TIF)

S5 Fig Impact of radial transport pathways on ^15^NH4+ accumulation in roots. ^15^N accumulation in roots of *tko*, *tko+1;2*, *tko+1;3*, *tko sgn3*, *tko sgn3+1;2*, and *tko sgn3+1;3* lines at 200 μM (A–B) or 4 mM (C–D) external ^15^NH_4_^+^. The estimated contribution of ATP or STP in absence or presence of an EB at 200 μM (B) or 4 mM ^15^NH_4_^+^ (D). Values were calculated by subtracting the background of *tko*. Plants were treated under the same conditions as in Fig 2B and 2C. Bars represent means ± SD (*n* = 4 biological replicates). Different letters indicate significant differences according to Tukey’s multiple test at *p* < 0.05. Underlying data can be found in S1 Data. ATP, apoplastic transport pathway; DW, dry weight; EB, endodermal bypass; N, nitrogen; ^15^NH_4_^+^, ^15^N-labeled ammonium; STP, symplastic transport pathway; *sgn3*, *schengen 3*; *tko*, *amt1;1amt1;2amt1;3*.
(TIF)

S6 Fig Relative transcript levels of *AMT1;1*, *AMT1;2*, and *AMT1;3* in roots of WT and *sgn3* mutant plants. Relative transcript levels were analyzed by real-time PCR using 3 reference genes: *Actin 2*, *ubiquitin 2*, and *ubiquitin 10*. Results were normalized by geNorm software. Since V_2/3_ < 0.15, the optimal number of internal control genes for this experiment was 2. Due to their lower variation, *Actin2* and *ubiquitin 10* were selected for normalization of AMT transcript abundance. Plants (WT, Columbia-0, *sgn3;3*, or *sgn3;4*) were grown hydroponically for 5 weeks in full nutrient solution containing 2 mM NO_3_^-^ as the sole N source and grown on N-free nutrient solution for 3 d before harvest (as in Fig 2). Bars represent means ± SD (*n* = 4–8 biological replicates). Transcript levels of *AMTs* in WT and 2 independent *sgn3* mutant lines were compared by Tukey’s multiple test at *p* < 0.05. Underlying data can be found in S1 Data. AMT, ammonium transporter; N, nitrogen; NO_3_^-^, nitrate; n.s., not significant; *sgn3*, *schengen 3*; WT, wild-type.
(TIF)

S7 Fig Shoot phenotype, potassium (K) and total nitrogen (N) concentrations in shoots of WT, *sgn3*, *tko*, and *tko sgn3* lines. (A) Phenotype of shoots at the time of sampling. Plants were grown hydroponically for 5 weeks in nutrient solution containing 2 mM NO_3_^-^ followed by 3 d of N starvation. Scale bars = 1 cm. The experiment was repeated 3 times and representative images are shown. (B and C) Concentrations of K (B) and total nitrogen (C) in shoots of WT (Columbia-0), *sgn3*, *tko*, and *tko sgn3* mutant lines. Bars represent means ± SD (*n* = 4 biological replicates). Different letters indicate significant differences according to Tukey’s multiple test at *p* < 0.05. Underlying data can be found in S1 Data. N, nitrogen; NO_3_^-^, nitrate; *sgn3*, *schengen 3*; *tko*, *amt1;1amt1;2amt1;3*; WT, wild-type.
(TIF)

S8 Fig Spatial distribution of AMT transporters and diffusion barriers along the primary root axis. (A-D) Colocalization of AMT1;2:GFP with CSs in mature (A), middle (B), and young (C) root zones. (E-H) Colocalization of AMT1;3:GFP with CS in mature (E), middle (F), and young (G) root zones. Magenta fluorescence shows penetration of PI into the apoplastic space of the stele (left panel). White arrows indicate blockage of PI by functional CS. Right panel shows AMT-dependent green fluorescence. Scale bars represent 50 μm. (D and H) Penetration of PI into the apoplastic space of the stele occurs from the root apex to the position marked by white arrows. (D) Localized expression of *proAMT1;2*-driven AMT1;2:GFP expands shootward approximately from the site where PI staining of the vasculature is blocked. (H) Localized expression of *proAMT1;3*-driven AMT1;3:GFP along the whole apical part of the primary root. Fluorescence of PI, GFP, and their overlay are shown by magenta, green, and white signals, respectively. (A–C and E–G) Enlarged root segments from young, middle, and mature zone according to (D and H). Underlying data can be found in S1 Data. AMT, ammonium transporter; CS, Casparian strip; GFP, green fluorescent protein; PI, propidium iodide.
(TIF)

S9 Fig Distribution of apoplastic barriers along the primary root axis in mutant lines. (A) Establishment of functional CSs, determined by the number of endodermal cells after onset of elongation, at which PI penetration to the stele is blocked. Continuous bars indicate that PI was not blocked up to >40 cells. (B and C) Quantification of the distribution of patchy suberin (B) or continuous suberin (C). Results show that only the formation of CSs was affected by the *sgn3* mutation, while suberization remained unaffected by expression of *SGN3* or *AMT1;3*. Bars represent means ± SD (*n* > 10 roots). No significant differences between lines were found according to one-way ANOVA; *p* < 0.05). Underlying data can be found in S1 Data. AMT, ammonium transporter; CS, Casparian strip; PI, propidium iodide; *sgn3*, *schengen 3*.
(TIF)

S1 Table Element concentrations in shoots of WT, *sgn3*, *tko*, and *tko sgn3* plants. Element concentrations were analyzed by (HR-ICP-MS). Values represent means ± SD (*n* = 4 biological replicates). Different letters indicate significant differences according to Tukey’s multiple test at *p* < 0.05. Underlying data can be found in S1 Data. HR-ICP-MS, high-resolution inductively coupled plasma mass spectrometry; *sgn3*, *schengen 3*; *tko*, *amt1;1amt1;2amt1;3*; WT, wild-type.
(DOCX)

S2 Table Relative contribution of individual pathways to radial ammonium transport in different zones along the primary root. Radial transport rates were calculated on the basis of normalized ^15^N shoot accumulation corresponding to values shown in Fig 2B–2E. (Underlying data can be found in S1 Data). The contribution of each pathway was calculated by subtracting the *tko* background value from transport rates of the corresponding reconstituted lines. “*tko* remainder” represents the remaining transport capacity, which is independent of 3 AMTs. *In the high-affinity range, *tko sgn3* and *tko* do not significantly differ. The contribution of the EB to radial transport is nearly 0. ^§^Assuming that all pathways are present at the same time. AMT, ammonium transporter; ATP, apoplastic transport pathway; EB, endodermal bypass; *sgn3*, *schengen 3*; STP, symplastic transport pathway; *tko*, *amt1;1amt1;2amt1;3*.
(DOCX)

S3 Table Primers used for genotyping and qPCR. All primer sequences are in 5′- to 3′-orientation.
(DOCX)

S1 Data (XLSX)

## Abbreviations

^15^NH_4_^+^: ^15^N-labeled ammonium
ABA: abscisic acid
AMT: ammonium transporter
ATP: apoplastic transport pathway
Ca^2+^: calcium ion
CIF: CS integrity factor
CO_2_: carbon dioxide
CS: Casparian strip
DW: dry weight
EB: endodermal bypass
ESB1: enhanced suberin 1
GFP: green fluorescent protein
GSO1: GASSHO1
ICP-MS: inductively coupled plasma mass spectrometry
K^+^: potassium ion
MEP: methylamine permease
MES: 2-(*N*-morpholino) ethanesulfonic acid
MYB36: myb domain protein 36
N: nitrogen
NH_4_^+^: ammonium
NO_3_^-^: nitrate
one-half MS: half-strength Murashige and Skoog basal salt mixture
PA: piperonylic acid
PI: propidium iodide
Rh-type: Rhesus-type
SGN3: schengen 3
Sr^2+^: strontium ion
STP: symplastic transport pathway
*tko*: *amt1;1amt1;2amt1;3*
WT: wild-type
