# Supplementary figures and images for: Root zone–specific localization of AMTs determines ammonium transport pathways and nitrogen allocation to shoots
Source: PLoS Biol. 2018 Oct 24;16(10):e2006024. doi: 10.1371/journal.pbio.2006024 (PMC6218093; doi:10.1371/journal.pbio.2006024)

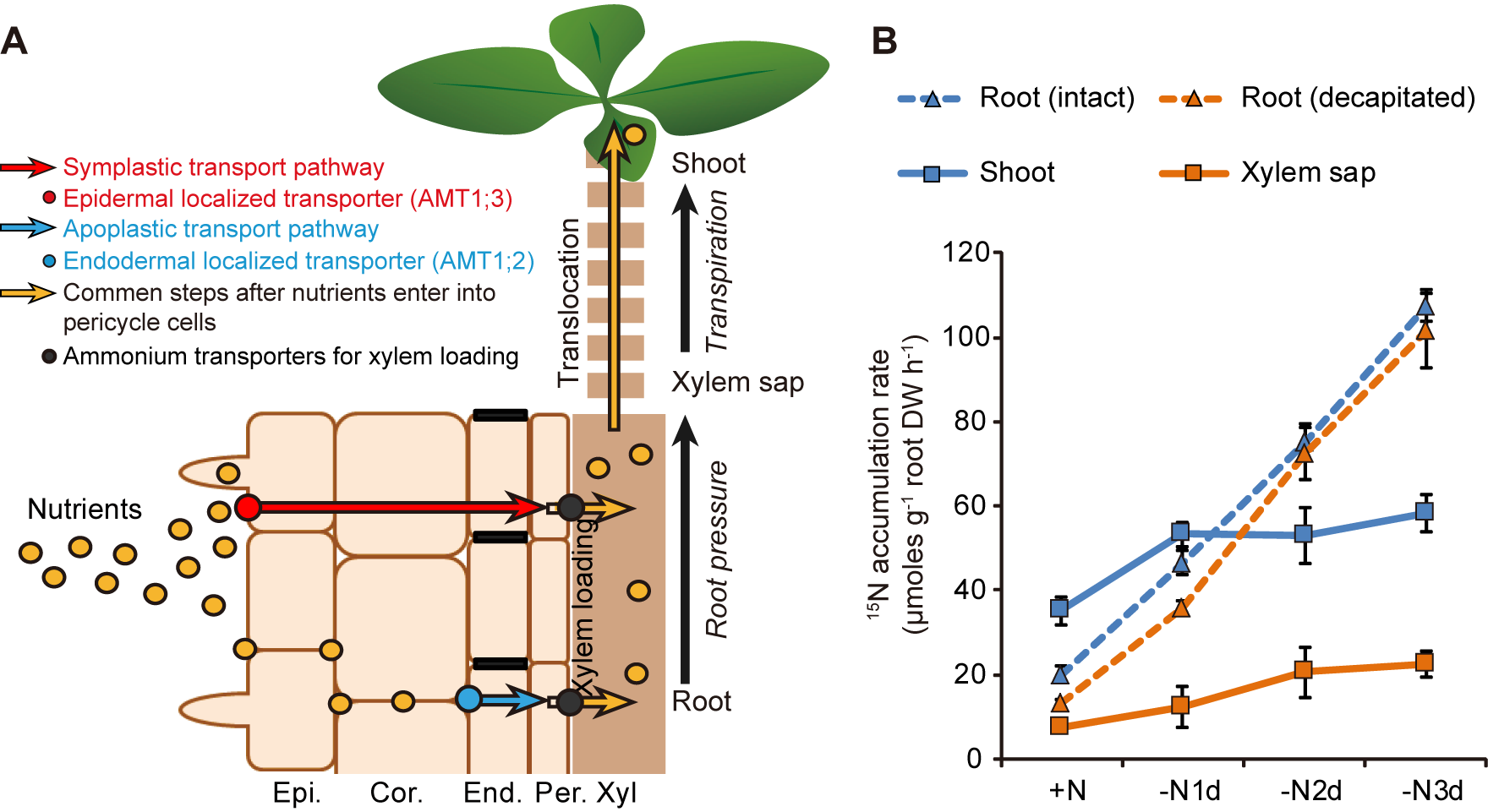

Supplement: S1 Fig — (A) Schematic representation of radial nutrient movement across the root and contribution of root pressure and transpiration to nutrient translocation to the shoot. (B) 15N accumulation rates in roots, shoots, and xylem sap of WT plants supplied with 200 μM 15NH4+. The data represent means ± SD (n = 4 biological replicates). Plants were grown hydroponically for 6 weeks in full nutrient solution followed by 0–3 d of N starvation to stepwise induce AMT1 transporters. 15N accumulation in roots from intact (dashed line, blue triangles) or decapitated (dashed line, orange triangle) plants increased steadily due to elevated expression of AMTs. Irrespective of whether radial transport rates were determined on the basis of 15N accumulation in the shoot (solid lines, blue squares) or in the xylem sap (solid lines, orange squares), radial transport rates leveled off, reflecting saturated xylem loading capacities. Relative to shoot 15N, xylem sap contained only one-third 15N, which was mostly likely due to the lack of transpiration as an additional driving force for root-to-shoot translocation of 15N. To properly reflect in planta conditions, 15N accumulation in shoots was considered as readout for root radial transport. Underlying data can be found in S1 Data. AMT, ammonium transporter; Cor., cortex; DW, dry weight; End., endodermis; Epi., epidermis; 15NH4+, 15N-labeled ammonium; N, nitrogen; Per. Pericycle; WT, wild-type; Xyl., xylem. (TIF) [file pbio.2006024.s001.tif]

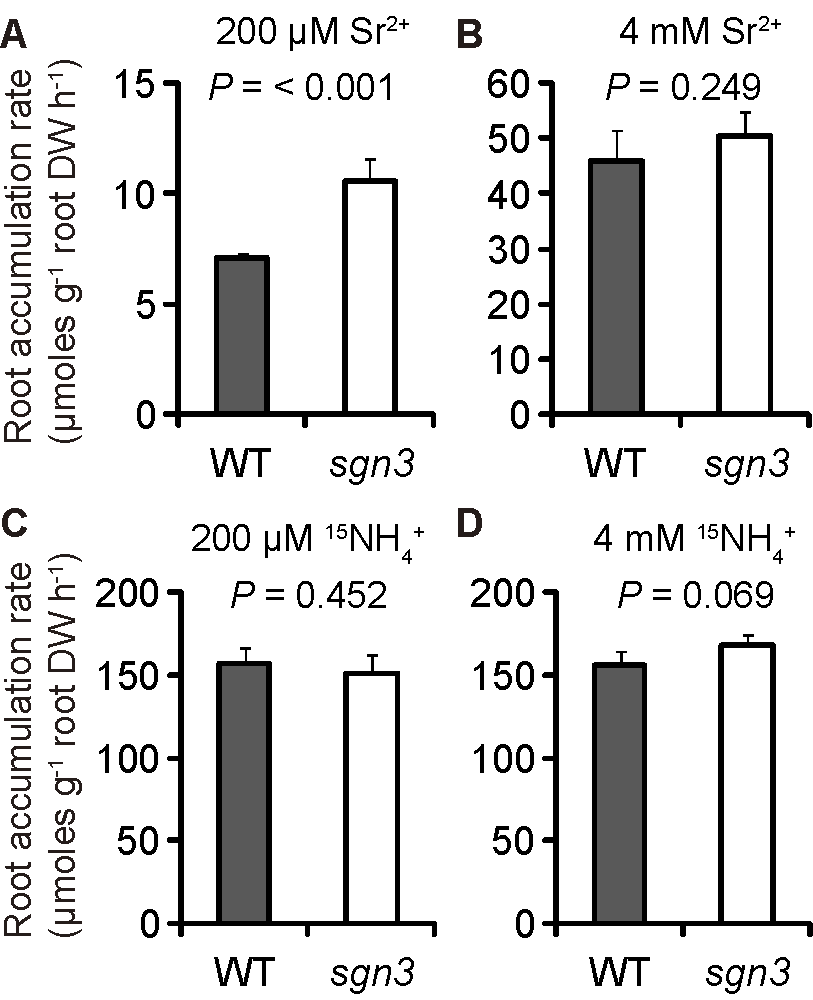

Supplement: S2 Fig — Accumulation of Sr2+ (A, B) or 15NH4+ (C, D) in roots of WT (Columbia-0) and sgn3 plants when supplied at an external concentration of 200 μM or 4 mM. Plants were grown under the same conditions as in Fig 1. Bars represent means ± SD. P values were calculated using Student t test (n = 4 biological replicates). Underlying data can be found in S1 Data. 15NH4+, 15N-labeled ammonium; sgn3, schengen 3; Sr2+, strontium ion; WT, wild-type. (TIF) [file pbio.2006024.s002.tif]

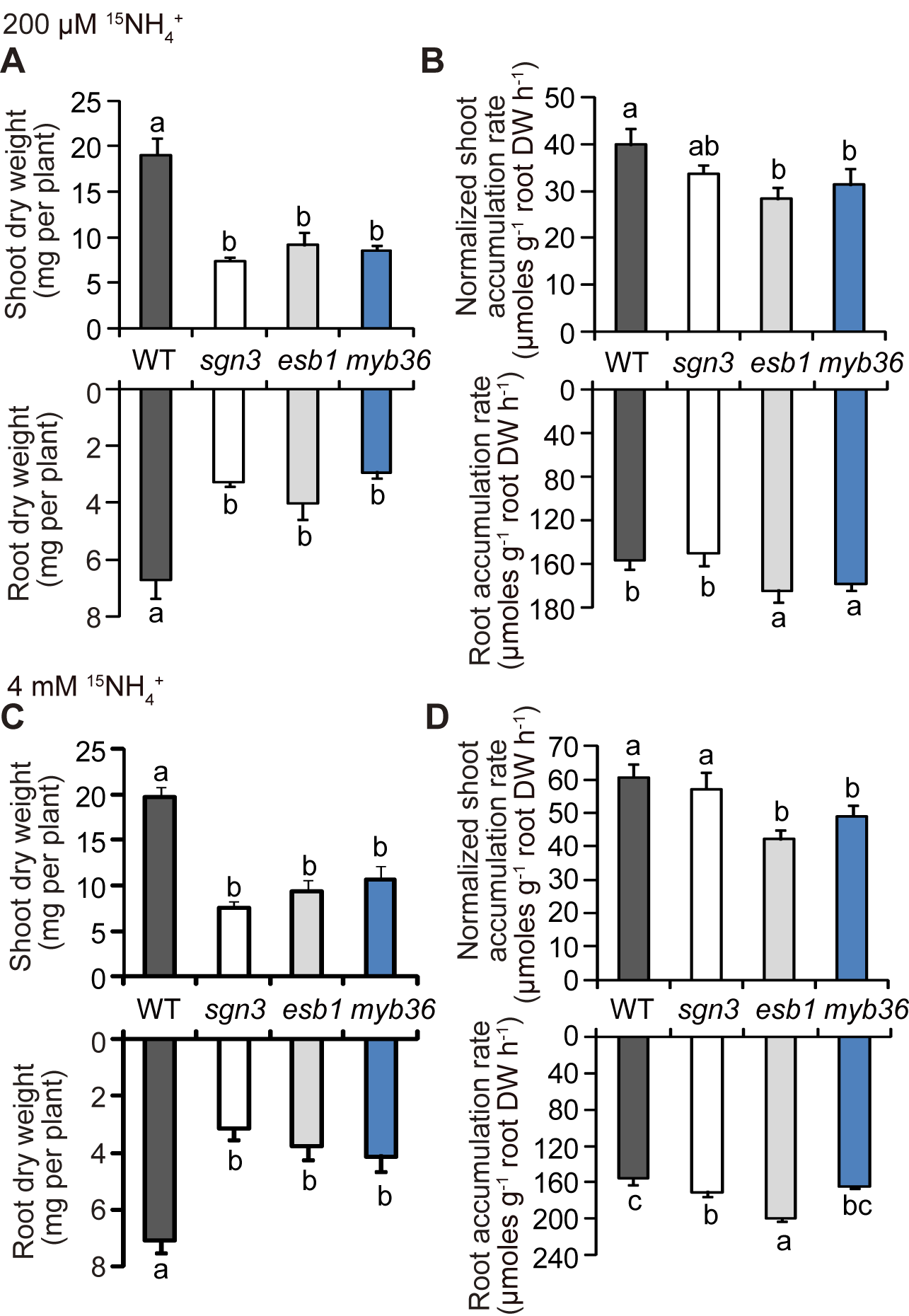

Supplement: S3 Fig — Shoot or root dry weights (A and C) and shoot or root accumulation (B and D) of 15NH4+ in WT (Columbia-0), sgn3, esb1, and myb36 mutant plants. Plants were exposed to 200 μM (A and B) or 4 mM (C and D) 15NH4+. Bars represent means ± SD (n = 4 biological replicates). Different letters indicate significant differences according to Tukey’s multiple test at p < 0.05. Underlying data can be found in S1 Data. 15NH4+, 15N-labeled ammonium; esb1, enhanced suberin 1; myb36, myb domain protein 36; sgn3, schengen 3; WT, wild-type. (TIF) [file pbio.2006024.s003.tif]

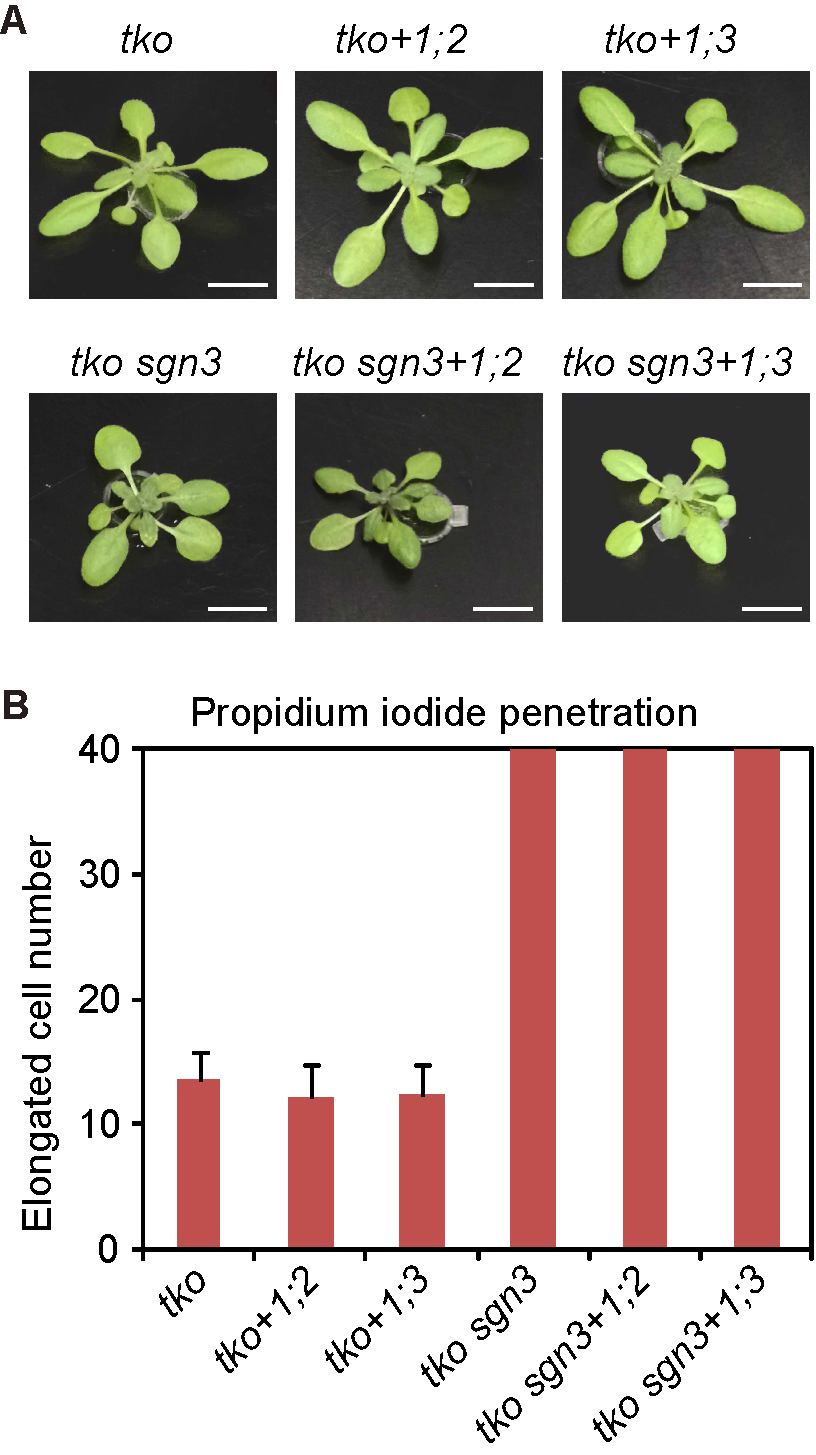

Supplement: S4 Fig — (A) Shoot growth of tko, tko+1;2, tko+1;3, tko sgn3, tko sgn3+1;2, and tko sgn3+1;3 mutant lines. All plants were grown hydroponically for 5 weeks in nutrient solution containing 2 mM NO3-, followed by 3 d of N starvation. Scale bars = 1 cm. The experiment was repeated 3 times, and representative images are shown. (B) Establishment of functional CSs was measured by counting at which elongated cell number the penetration of PI into the apoplastic space of the stele was blocked. Roots from 8-d-old agar-grown plants were stained with PI for 10 min. Bars represent means ± SD. At least 10 roots per mutant line were assessed. Continuous bars indicate that PI was not blocked up to >40 cells. Underlying data can be found in S1 Data. CS, Casparian strip; N, nitrogen; NO3-, nitrate; PI, propidium iodide; sgn3, schengen 3; tko, amt1;1amt1;2amt1;3. (TIF) [file pbio.2006024.s004.tif]

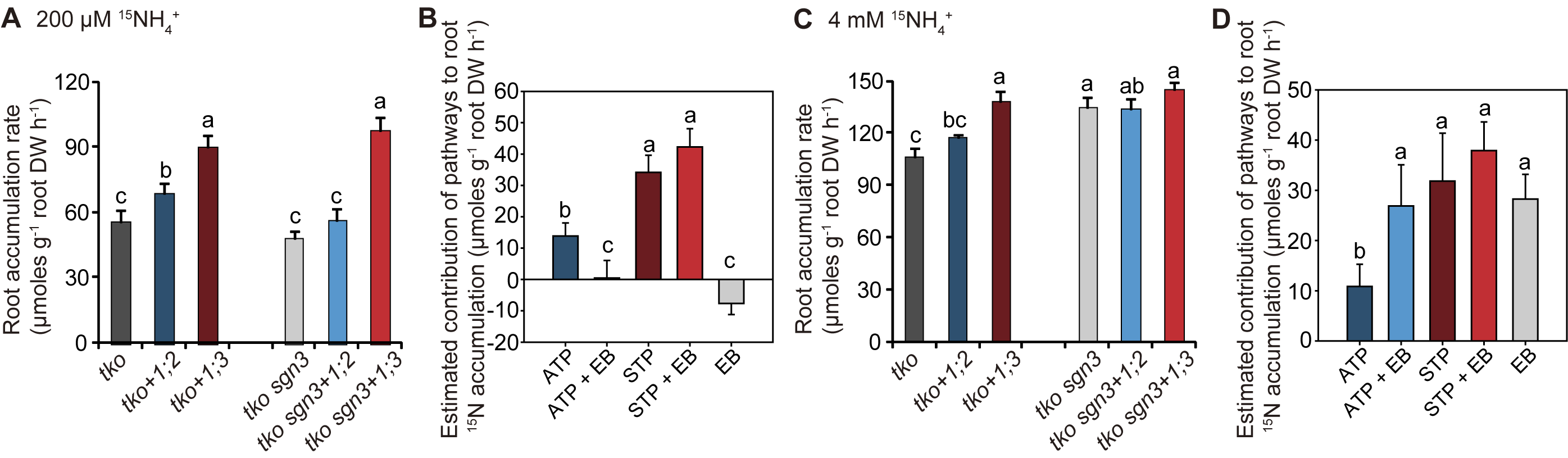

Supplement: S5 Fig — 15N accumulation in roots of tko, tko+1;2, tko+1;3, tko sgn3, tko sgn3+1;2, and tko sgn3+1;3 lines at 200 μM (A–B) or 4 mM (C–D) external 15NH4+. The estimated contribution of ATP or STP in absence or presence of an EB at 200 μM (B) or 4 mM 15NH4+ (D). Values were calculated by subtracting the background of tko. Plants were treated under the same conditions as in Fig 2B and 2C. Bars represent means ± SD (n = 4 biological replicates). Different letters indicate significant differences according to Tukey’s multiple test at p < 0.05. Underlying data can be found in S1 Data. ATP, apoplastic transport pathway; DW, dry weight; EB, endodermal bypass; N, nitrogen; 15NH4+, 15N-labeled ammonium; STP, symplastic transport pathway; sgn3, schengen 3; tko, amt1;1amt1;2amt1;3. (TIF) [file pbio.2006024.s005.tif]

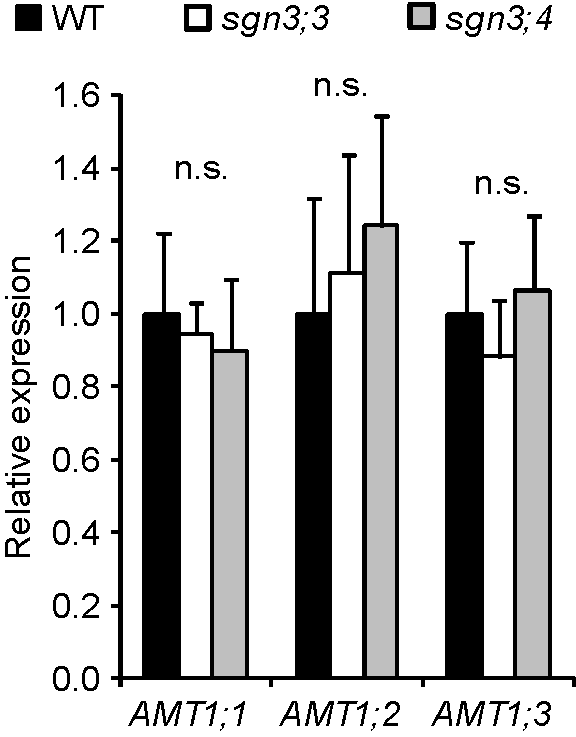

Supplement: S6 Fig — Relative transcript levels were analyzed by real-time PCR using 3 reference genes: Actin 2, ubiquitin 2, and ubiquitin 10. Results were normalized by geNorm software. Since V2/3 < 0.15, the optimal number of internal control genes for this experiment was 2. Due to their lower variation, Actin2 and ubiquitin 10 were selected for normalization of AMT transcript abundance. Plants (WT, Columbia-0, sgn3;3, or sgn3;4) were grown hydroponically for 5 weeks in full nutrient solution containing 2 mM NO3- as the sole N source and grown on N-free nutrient solution for 3 d before harvest (as in Fig 2). Bars represent means ± SD (n = 4–8 biological replicates). Transcript levels of AMTs in WT and 2 independent sgn3 mutant lines were compared by Tukey’s multiple test at p < 0.05. Underlying data can be found in S1 Data. AMT, ammonium transporter; N, nitrogen; NO3-, nitrate; n.s., not significant; sgn3, schengen 3; WT, wild-type. (TIF) [file pbio.2006024.s006.tif]

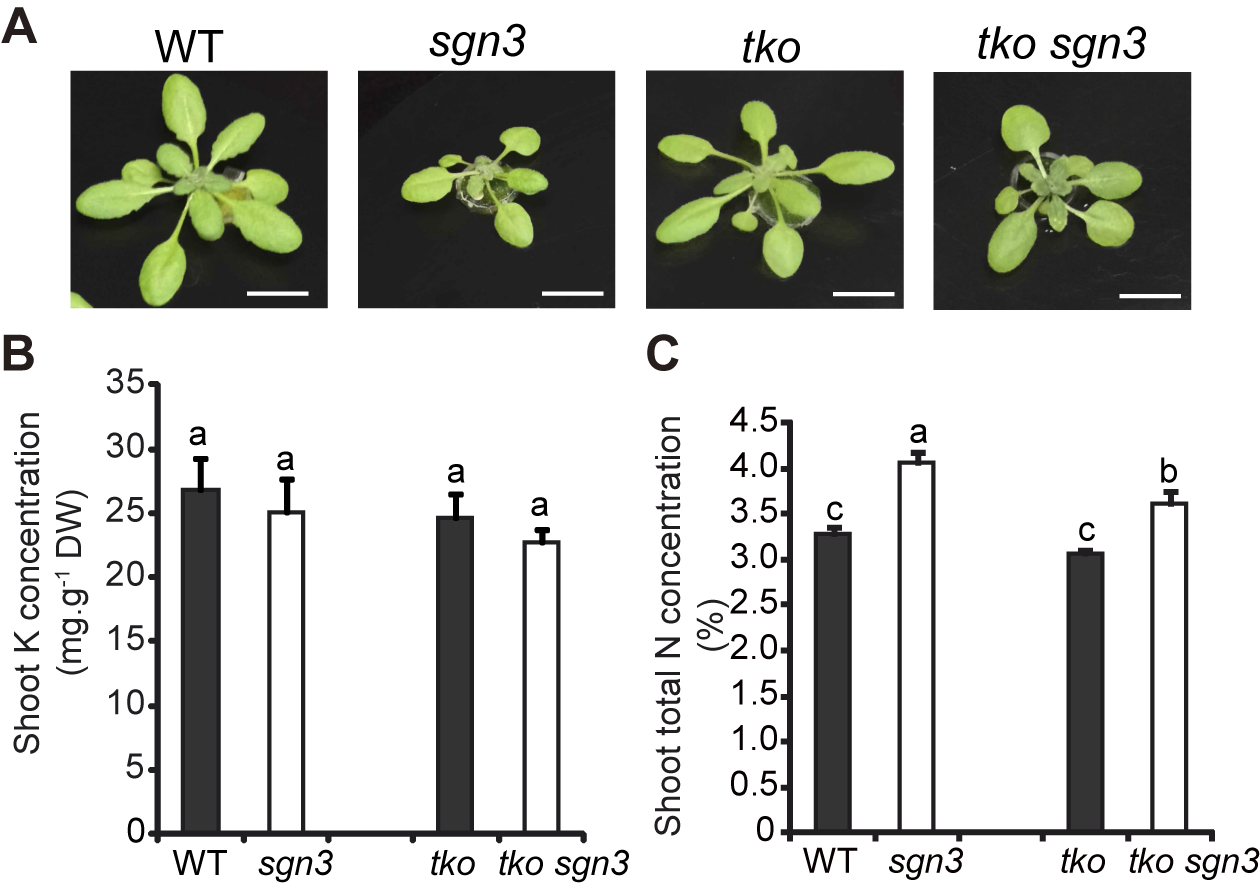

Supplement: S7 Fig — (A) Phenotype of shoots at the time of sampling. Plants were grown hydroponically for 5 weeks in nutrient solution containing 2 mM NO3- followed by 3 d of N starvation. Scale bars = 1 cm. The experiment was repeated 3 times and representative images are shown. (B and C) Concentrations of K (B) and total nitrogen (C) in shoots of WT (Columbia-0), sgn3, tko, and tko sgn3 mutant lines. Bars represent means ± SD (n = 4 biological replicates). Different letters indicate significant differences according to Tukey’s multiple test at p < 0.05. Underlying data can be found in S1 Data. N, nitrogen; NO3-, nitrate; sgn3, schengen 3; tko, amt1;1amt1;2amt1;3; WT, wild-type. (TIF) [file pbio.2006024.s007.tif]

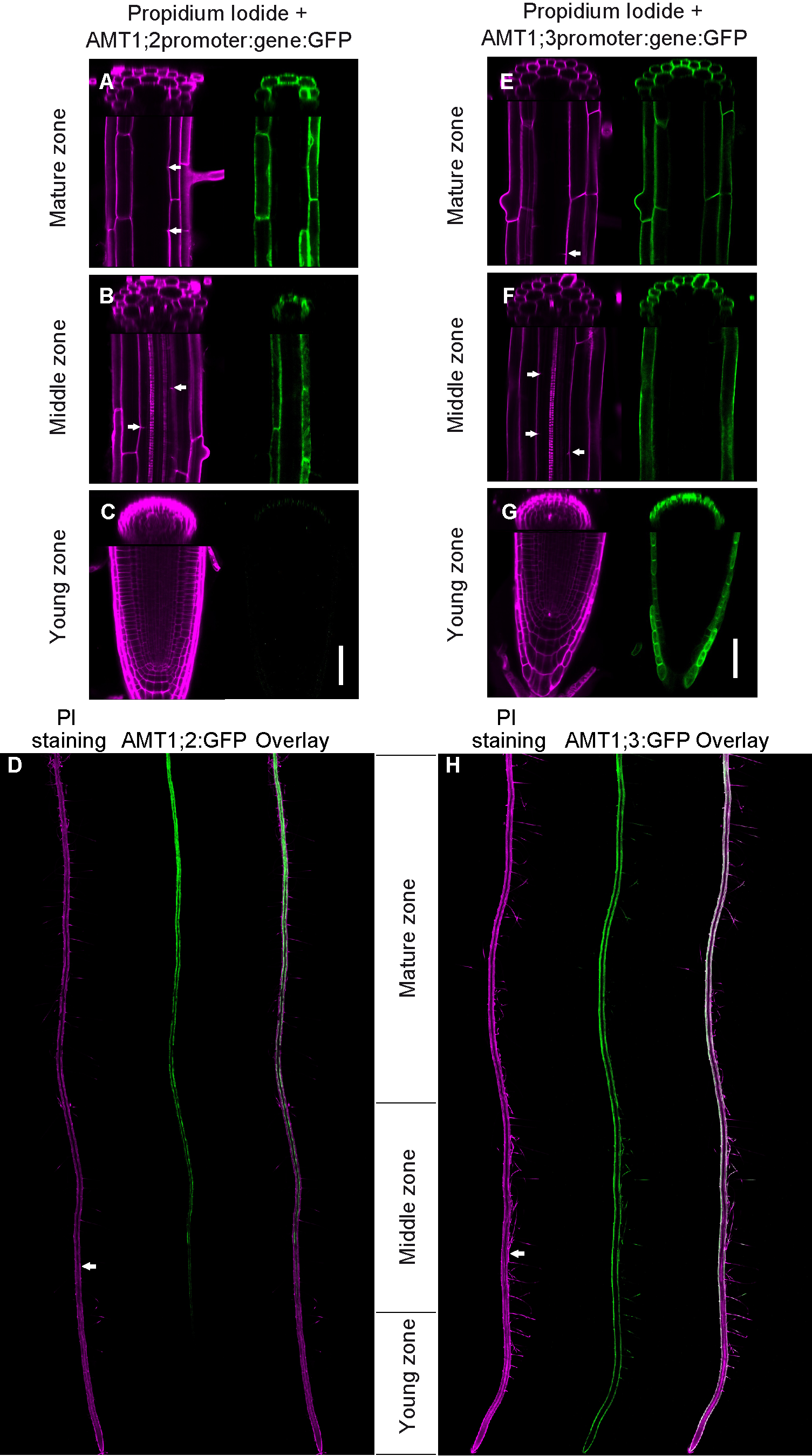

Supplement: S8 Fig — (A-D) Colocalization of AMT1;2:GFP with CSs in mature (A), middle (B), and young (C) root zones. (E-H) Colocalization of AMT1;3:GFP with CS in mature (E), middle (F), and young (G) root zones. Magenta fluorescence shows penetration of PI into the apoplastic space of the stele (left panel). White arrows indicate blockage of PI by functional CS. Right panel shows AMT-dependent green fluorescence. Scale bars represent 50 μm. (D and H) Penetration of PI into the apoplastic space of the stele occurs from the root apex to the position marked by white arrows. (D) Localized expression of proAMT1;2-driven AMT1;2:GFP expands shootward approximately from the site where PI staining of the vasculature is blocked. (H) Localized expression of proAMT1;3-driven AMT1;3:GFP along the whole apical part of the primary root. Fluorescence of PI, GFP, and their overlay are shown by magenta, green, and white signals, respectively. (A–C and E–G) Enlarged root segments from young, middle, and mature zone according to (D and H). Underlying data can be found in S1 Data. AMT, ammonium transporter; CS, Casparian strip; GFP, green fluorescent protein; PI, propidium iodide. (TIF) [file pbio.2006024.s008.tif]

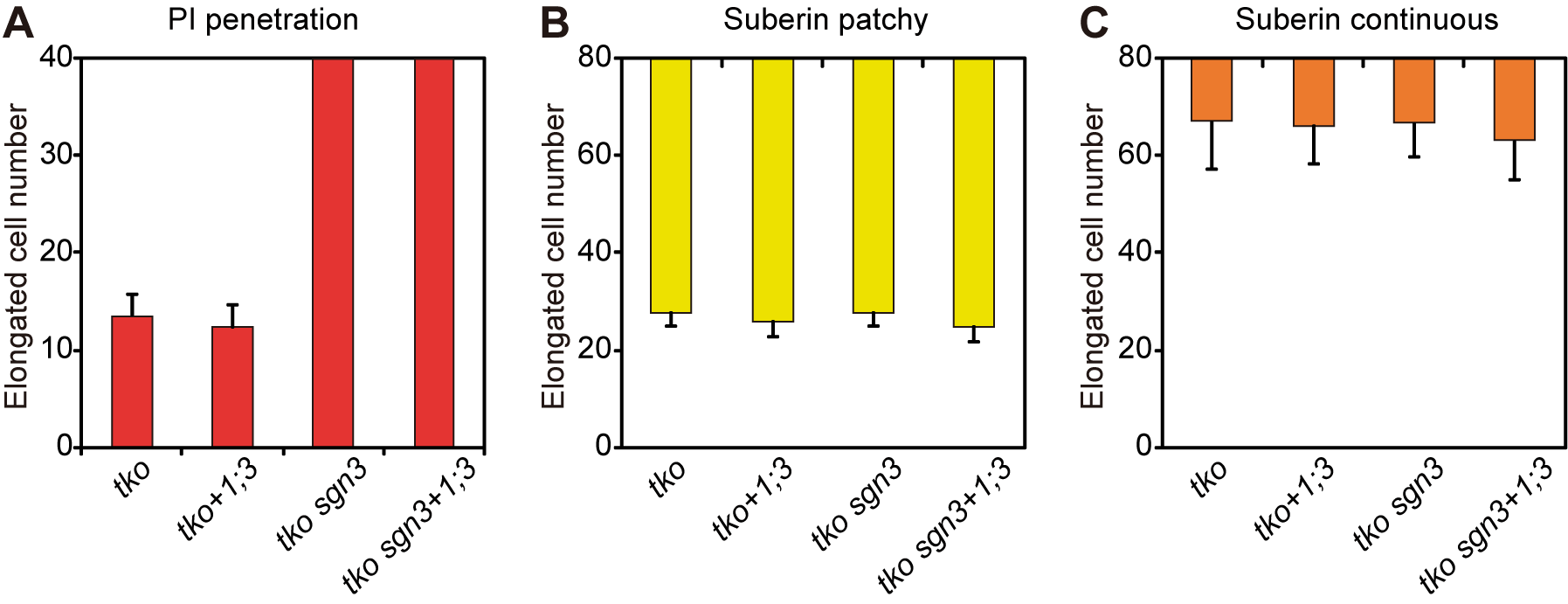

Supplement: S9 Fig — (A) Establishment of functional CSs, determined by the number of endodermal cells after onset of elongation, at which PI penetration to the stele is blocked. Continuous bars indicate that PI was not blocked up to >40 cells. (B and C) Quantification of the distribution of patchy suberin (B) or continuous suberin (C). Results show that only the formation of CSs was affected by the sgn3 mutation, while suberization remained unaffected by expression of SGN3 or AMT1;3. Bars represent means ± SD (n > 10 roots). No significant differences between lines were found according to one-way ANOVA; p < 0.05). Underlying data can be found in S1 Data. AMT, ammonium transporter; CS, Casparian strip; PI, propidium iodide; sgn3, schengen 3. (TIF) [file pbio.2006024.s009.tif]
