# Supplementary material for: Root zone–specific localization of AMTs determines ammonium transport pathways and nitrogen allocation to shoots
Source: PLoS Biol. 2018 Oct 24;16(10):e2006024. doi: 10.1371/journal.pbio.2006024 (PMC6218093; doi:10.1371/journal.pbio.2006024)
Supplement: S1 Table — Element concentrations were analyzed by (HR-ICP-MS). Values represent means ± SD (n = 4 biological replicates). Different letters indicate significant differences according to Tukey’s multiple test at p < 0.05. Underlying data can be found in S1 Data. HR-ICP-MS, high-resolution inductively coupled plasma mass spectrometry; sgn3, schengen 3; tko, amt1;1amt1;2amt1;3; WT, wild-type. (DOCX) [file pbio.2006024.s010.docx]

| **Element** | **Wild-type (WT)** | ***sgn3*** | ***tko*** | ***tko sgn3*** |
| --- | --- | --- | --- | --- |
|  | **µg g^-1^ dry weight** | **µg g^-1^ dry weight** | **µg g^-1^ dry weight** | **µg g^-1^ dry weight** |
| K | 26768.89 ± 2525.70 | 25046.45 ± 2642.16 | 24557.94 ± 1986.20 | 22649.86 ± 1043.01 |
| Ca | 13994.41 ± 2447.00 | 10813.36 ± 1937.11 | 12601.60 ± 853.65 | 10323.94 ± 2025.38 |
| Fe | 77.5 ± 12.82 bc | 115.35 ± 7.32 a | 66.20 ± 3.30 c | 95.53 ± 7.97 b |
| Zn | 97.32 ± 7.72 a | 50.88 ± 1.65 c | 70.15 ± 7.17 b | 45.66 ± 2.16 c |
| Mn | 299.58 ± 17.25 a | 266.89 ± 22.14 ab | 302.67 ± 22.66 a | 247.52 ± 9.96 b |
