# Supplementary material for: Root zone–specific localization of AMTs determines ammonium transport pathways and nitrogen allocation to shoots
Source: PLoS Biol. 2018 Oct 24;16(10):e2006024. doi: 10.1371/journal.pbio.2006024 (PMC6218093; doi:10.1371/journal.pbio.2006024)
Supplement: S2 Table — Radial transport rates were calculated on the basis of normalized 15N shoot accumulation corresponding to values shown in Fig 2B–2E. (Underlying data can be found in S1 Data). The contribution of each pathway was calculated by subtracting the tko background value from transport rates of the corresponding reconstituted lines. “tko remainder” represents the remaining transport capacity, which is independent of 3 AMTs. *In the high-affinity range, tko sgn3 and tko do not significantly differ. The contribution of the EB to radial transport is nearly 0. §Assuming that all pathways are present at the same time. AMT, ammonium transporter; ATP, apoplastic transport pathway; EB, endodermal bypass; sgn3, schengen 3; STP, symplastic transport pathway; tko, amt1;1amt1;2amt1;3. (DOCX) [file pbio.2006024.s011.docx]

| **Genotype** | **Pathway** | **Radial transport rate (µmoles g^-1^ root DW h^-1^)** | **Contribution by each pathway (µmoles g^-1^ root DW h^-1^)** | **Contribution by all pathways** | **Relative contribution of each pathway^§^ (%)** | **Root zone** |
| --- | --- | --- | --- | --- | --- | --- |
| **200 µM external ^15^NH_4_^+^** | | | | | | |
| *tko+AMT1;3* | **tko + STP** | 30.69 | 20.60 | 46.14 | 44.6 | Zone 2 |
| *tko+AMT1;2* | **tko + ATP** | 25.54 | 15.45 |  | 33.5 |  |
| *tko* | **tko remainder** | 10.09 | 10.09 |  | 21.9 |  |
| *tko sgn3* | **tko + EB** | 7.33 | 0* | 49.97 | 0 | Zone 1 |
| *tko sgn3 +AMT1;2* | **tko + EB + ATP** | 17.85 | 7.76 |  | 15.5 |  |
| *tko sgn3 +AMT1;3* | **tko + EB + STP** | 42.21 | 32.12 |  | 64.3 |  |
| *tko* | **tko remainder** | 10.09 | 10.09 |  | 20.2 |  |
| **4 mM external ^15^NH_4_^+^** | | | | | | |
| *tko+AMT1;3* | **tko + STP** | 56.43 | 11.86 | 81.11 | 14.6 | Zone 2 |
| *tko+AMT1;2* | **tko + ATP** | 69.25 | 24.68 |  | 30.4 |  |
| *tko* | **tko remainder** | 44.57 | 44.57 |  | 55.0 |  |
| *tko sgn3* | **tko + EB** | 72.36 | 27.79 | 127.31 | 21.8 | Zone 1 |
| *tko sgn3 +AMT1;2* | **tko + EB + ATP** | 62.84 | 18.27 |  | 14.4 |  |
| *tko sgn3 +AMT1;3* | **tko + EB + STP** | 81.25 | 36.68 |  | 28.8 |  |
| *tko* | **tko remainder** | 44.57 | 44.57 |  | 35.0 |  |
