# Supplementary material for: Root zone–specific localization of AMTs determines ammonium transport pathways and nitrogen allocation to shoots
Source: PLoS Biol. 2018 Oct 24;16(10):e2006024. doi: 10.1371/journal.pbio.2006024 (PMC6218093; doi:10.1371/journal.pbio.2006024)
Supplement: S3 Table — All primer sequences are in 5′- to 3′-orientation. (DOCX) [file pbio.2006024.s012.docx]

| **Genotyping primers (co-dominant markers)** | | |
| --- | --- | --- |
| Gene name | Gene number | Primer sequence |
| *AMT1;1* | *AT4G13510* | 11RB: GAATGGCGAATGAGACCTCAATTGCGAG |
|  |  | 11F: GAAATTCGAAATTTCGCTGCCATTTCC |
|  |  | 11R: GGTGCTGAAAAGCAAATCTCCATCG |
| *AMT1;2* | *AT1G64780* | 12SPM: GCTTGTTGAACCGACACTTTAACATAAG |
|  |  | 12F: CTTACTACCTCTTCGGATTCGCATTC |
|  |  | 12R: GTTAACAGTTCCACACTATCTGTCC |
| *AMT1;3* | *AT3G24300* | 13RB: TCCTTCAATCGTTGCGGTTCTGTCAGTTC |
|  |  | 13F: TTGGACATATCTAAGAGTTAAGGGTG |
|  |  | 13R: AACCGGGTAAACAAATCCGGTTAAGA |
| *SGN3;3* | *AT4G20140* | sgn33LBa1: TGGTTCACGTAGTGGGCCATCG |
|  |  | sgn33F: CGGTTTAGGTCAACCGGGTATCATC |
|  |  | sgn33R: ACTGGCGGGAAGTCCACCCACAA |
|  |  |  |
| **qPCR primers** |  |  |
| Gene name | Gene number | Primer sequence |
| *AMT1;1* | *AT4G13510* | qPCR11F: ATGAAATTGTTGCGGATATCGTCC |
|  |  | qPCR11R: TCTCCTAAGCTGAATGGCTTTGTG |
| *AMT1;2* | *AT1G64780* | qPCR12F: TCGACTCCTACACCGACCTT |
|  |  | qPCR12R: GTCCAACATGTTTGGTGCCC |
| *AMT1;3* | *AT3G24300* | qPCR13F: TCATGGCTTCTGTCGTCCTTATCGG |
|  |  | qPCR13R: TAAACGCGAGGAGGAGTAGCTGATC |
| *AtActin2* | *AT3G18780* | ActinF: TCGGTGGTTCCATTCTTGCT |
|  |  | ActinR: GCTTTTTAAGCCTTTGATCTTGAGAG |
| *UBQ10* | *AT4G05320* | qUBQ10-F: CTTCGTCAAGACTTTGACCG |
|  |  | qUBQ10-R: CTTCTTAAGCATAACAGAGACGAG |
| *UBQ2* | *AT2G36170* | qUBQ2F: CCAAGATCCAGGACAAAGAAGGA |
|  |  | qUBQ2R: TGGAGACGAGCATAACACTTGC |
